# A meta-analysis of unilateral axillary approach for robotic surgery compared with open surgery for differentiated thyroid carcinoma

**DOI:** 10.1371/journal.pone.0298153

**Published:** 2024-04-11

**Authors:** Xinjun Zhang, Junkang Yu, Jinhui Zhu, Haibo Wei, Ning Meng, Mingrong Hu, Jingjie Tang

**Affiliations:** 1 Department of Thyroid and Breast Surgery, Affiliated Hospital of Hangzhou Normal University, Hangzhou, Zhejiang Province, China; 2 Institute of Bioengineering and Medical Engineering, Guangdong Academy of Sciences, Guangzhou, Guangdong Province, China; Public Library of Science, UNITED KINGDOM

## Abstract

**Objective:**

The Da Vinci Robot is the most advanced micro-control system in endoscopic surgical instruments and has gained a lot of valuable experience today. However, the technical feasibility and oncological safety of the robot over open surgery are still uncertain. This work is to systematically evaluate the efficacy of the unilateral axillary approach for robotic surgery compared to open surgery for differentiated thyroid carcinoma.

**Methods:**

PubMed, Embase, Cochrane Library, and Web of Science databases were utilized to search for relevant literatures of robotic thyroid surgery using unilateral axillary approach compared to open thyroid surgery, and a meta-analysis was performed using RevMan software version 5.3. Statistical analysis was performed through Mantle-Haenszel and inverse variance methods.

**Results:**

Twelve studies with a total of 2660 patients were included in the meta-analysis. The results showed that compared with the open group, the robotic group had a longer total thyroidectomy time, shorter hospital stay, less intraoperative bleeding, more postoperative drainage, fewer retrieved central lymph nodes, and higher cosmetic satisfaction (all *P* < 0.05). In contrast, temporary and permanent laryngeal recurrent nerve injury, temporary and permanent hypoparathyroidism or hypocalcemia, brachial plexus nerve injury, number of retrieved central lymph nodes, number of retrieved lymph nodes in the lateral cervical region, number of lymph node metastases in the lateral cervical region, hematoma, seroma, lymphatic leak, stimulated thyroglobulin (sTg) and unstimulated thyroglobulin (uTg), and the number and recurrence rate of patients with sTg <1ng/ml were not statistically different between the two groups (*P* > 0.05).

**Conclusions:**

The unilateral axillary approach for robotic thyroid surgery may achieve outcomes similar to those of open surgery. Further validation is required in a prospective randomized controlled trial.

## 1 Introduction

Thyroid cancer is one of the most common endocrine-related cancers, and papillary and follicular carcinomas are collectively referred to as differentiated thyroid carcinomas (DTC). Compared to open thyroidectomy (OT), endoscopic thyroidectomy (ET) avoids neck scarring and has excellent cosmetic results, making it popular among patients. However, the scope of ET is affected by the limitations of rigid instruments. Robotic thyroidectomy (RT), which has the advantage of both "flexible" robotic arms and aesthetic appearance of the neck, is expected to overcome the shortcomings of ET and be gradually carried out all around the world. Currently, several approaches to RT have been used in clinical practice, including the unilateral axillary, bilateral axillary breast, transoral, and retroauricular approaches [1]. Because of the small number of cases in each study, the efficacy cannot be objectively evaluated, and most of the existing meta-analyses have covered different approaches, resulting in high heterogeneity and affecting the credibility of conclusions [2–4]. Therefore, this study intends to conduct a meta-analysis of RT versus OT in a single approach (unilateral axillary approach), and systematically evaluate their surgical efficacy as below.

## 2 Materials and methods

### 2.1 Literature search

PubMed, Embase, Cochrane Library, and Web of Science databases were used for clinical literature research on the comparison of RT and OT. Keywords used were "robotic", "open", "conventional", "thyroidectomy", and "neoplasms". The retrieval time is from the establishment of the database to April 2023. The search strategy and PICOS strategy are shown in S1 Table.

### 2.2 Inclusion criteria

(1) randomized, non-randomized, case-control, cohort, and cross-sectional studies of RT and OT; (2) the surgical approach of RT is limited to the unilateral axillary approach; (3) postoperative pathology of patients confirmed to be DTC; and (4) at least one surgery-related outcome indicator can be provided.

### 2.3 Exclusion criteria

(1) history of neck surgery or radioactive iodine therapy; (2) repeated publication of literature, case reports, conference papers, expert comments, reviews, etc.; and (3) lack of original data and fruitless contact with authors.

### 2.4 Data extraction and literature quality evaluation

Two researchers independently screened the literature and extracted the relevant information. In case of disagreement, a third researcher was solicited to make the final decision. The extracted data included the following terms: basic information of the included studies (including the author, publication year, country, number of surgeries, sex, age, surgery procedure, follow-up time, study types, and quality scores) and 21 outcomes related to surgery (including temporary and permanent recurrent laryngeal nerve injury, temporary and permanent hypoparathyroidism or hypocalcemia, brachial plexus injury, number of retrieved central lymph nodes, number of central lymph node metastases, number of dissected lymph nodes in the lateral neck region, number of metastatic lymph nodes in the lateral neck region, time of total thyroidectomy, length of hospital stay, intraoperative blood loss, postoperative drainage, hematoma, seroma, lymphatic leakage, level of stimulated thyroglobulin (sTg) and unstimulated thyroglobulin (uTg), number of people with sTg <1ng/ml, recurrence rate, and cosmetic satisfaction rate). The criteria for temporary/permanent recurrent laryngeal nerve injury, temporary/permanent hypoparathyroidism, and hypocalcemia were determined according to the duration of each study. The rate of recurrent laryngeal nerve injury was calculated as follows: number of recurrent laryngeal nerve injuries/total number of recurrent laryngeal nerve exposure ×100%. The sTg levels were compared only in patients treated with radioactive iodine after total thyroidectomy. Cochrane criteria, Methodological Index for Non-Randomized Studies(MINORS), Newcastle-Ottawa scale(NOS), and Healthcare Research and Quality(AHRQ) were used to evaluate the quality of literature for randomized controlled studies, non-randomized controlled studies, cohort studies, and cross-sectional studies, respectively.

### 2.5 Statistical methods

RevMan software (version 5.3) was used for the meta-analysis. Statistical analysis was performed through Mantle-Haenszel and inverse variance methods. The risk ratio (RR) was used as the effect indicator for dichotomous variables, and the weighted mean difference (MD) or standardized mean difference was used for continuous variables. Random-effects models were used, with estimates and 95% confidence intervals (CIs) provided for all indicators. The test level for the meta-analysis was α = 0.05, and *P* < 0.05 was set as statistically significant.

### 2.6 Patient and public involvement

None.

### 2.7 Ethics approval statement

All analyses were based on previous published studies, thus no ethical approval and patient consent are required.

## 3 Results

### 3.1 Literature retrieval results

**Fig 1:**
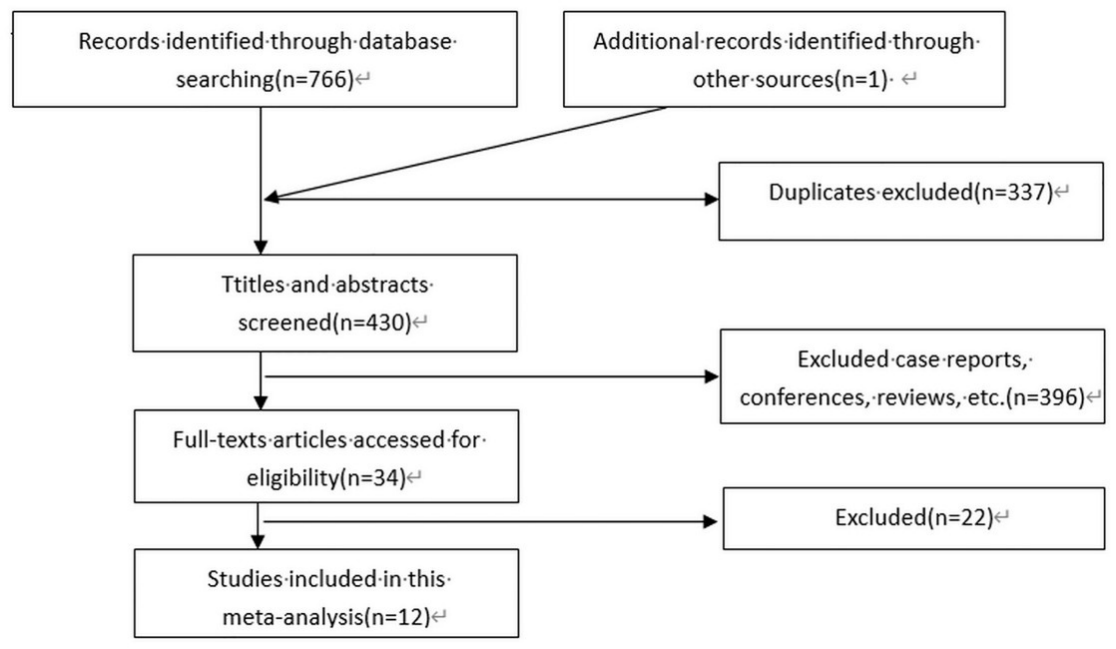
Flow diagram of the study selection.

After literature searching, 766 articles were initially retrieved, one article was added by tracing the original text through the references, 337 duplicate articles were removed, 396 were screened out by reading the titles and abstracts, 22 articles were excluded in strict accordance with the inclusion and exclusion criteria, and 12 studies were finally included in this meta-analysis. A flowchart of the search process is shown in Fig 1.

### 3.2 Basic characteristics and quality assessment of the included studies

A total of 12 publications were included in this meta-analysis [5–16], including three non-randomized controlled studies, which had a MINORS score of 17,21,20 separately [5, 7, 9], eight retrospective case-cohort studies, which had a NOS score of 7,8,8,8,9,9,8,9 separately [6, 8, 11–16], and one cross-sectional study, which had a AHRQ score of 18 [10]. In addition, 10 of the articles were from Korea [5–7, 9–14, 16] and 2 from the United States [8, 15]. There were a total of 2660 patients (1208 in the RT group and 1452 in the OT group), among which the proportion of female patients was 76% in the RT group and 72% in the OT group, while the proportion of total thyroidectomy was 65% in the RT group and 77% in the OT group (Tables 1 and 2). Whereas 22 articles excluded after reviewing the full text is shown in Table 3 [17–38].

**Table 1:** Characteristics of the included 12 studies (part 1).

First Author	Year	Country	Number	Sex (F/M)		Age (mean, year)	
			(RT/OT)	RT	OT	RT	OT
Lee [5]	2010	Korea	41/43	38/3	40/3	39.0	37.7
Kang [6]	2012	Korea	56/109	46/10	83/26	35.8	46.1
Lee [7]	2013	Korea	62/66	57/5	54/12	40.2	45.1
Noureldine [8]	2013	USA	24/35	20/4	21/14	45.4	52.6
Ryu [9]	2013	Korea	45/45	42/3	36/9	39.0	48.9
Song [10]	2014	Korea	44/67	38/6	54/13	41.9	51.5
Lee [11]	2016	Korea	206/206	24/182	25/181	41.1	42.1
Song [12]	2016	Korea	25/41	24/1	30/11	36.7	47.5
Tae [13]	2016	Korea	185/185	166/19	165/20	41.8	42.6
Kim [14]	2017	Korea	41/102	32/9	77/25	42.3	44.3
Garstka [15]	2018	USA	35/109	34/1	79/30	42.1	52.7
Cho [16]	2019	Korea	444/444	401/43	392/52	41.3	41.3
Total			1208/1452	922/286	1056/396		

**Table 2:** Characteristics of the included 12 studies (part 2).

First Author	Surgery		Level of lymph node dissection	Follow-up	Study design	Quality score
	RT	OT		(Months)		
Lee [5]	TT/STT(26/15)	TT/STT(26/17)	CCND	3	NRS	17
Kang [6]	TT(56)	TT(109)	MRND	12	Cohort	7
Lee [7]	TT(62)	TT(66)	MRND	12	NRS	21
Noureldine [8]	TT/Lob(17/7)	TT/Lob(27/8)	NR	12±2.2	Cohort	8
Ryu [9]	TT(45)	TT(45)	CCND	-	NRS	20
Song [10]	TT/Lob(37/7)	TT/Lob(59/8)	CCND	12	CSS	18
Lee [11]	TT(206)	TT(206)	CCND	74.2	Cohort	8
Song [12]	TT(25)	TT(41)	MRND	RT: 29 OT:34.8	Cohort	8
Tae [13]	TT/Lob(103/82)	TT/Lob(111/74)	CCND	43.6	Cohort	9
Kim [14]	TT(41)	TT(102)	MRND	66.6	Cohort	9
Garstka [15]	TT/Lob(13/22)	TT/Lob(73/36)	MRND	RT: 7.3±6.1 OT:9.9±8.8	Cohort	8
Cho [16]	TT/Lob(158/286)	TT/Lob(251/193)	CCND	28–99	Cohort	9
Total	TT:789	TT:1116				

TT, total thyroidectomy; STT, subtotal thyroidectomy; Lob, lobetomy; CCND, central component lymph node dissection; MRND, modified radical lymph node dissection; NRS, non-randomized-controlled study; CSS, cross-sectional study.

**Table 3:** 22 articles excluded after reviewing the full text.

First author	Year	Country	Main Reason for Exclusion
Tae [17]	2011	Korea	Pathology containing non-DTC
Foley [18]	2012	USA	Pathology containing non-DTC
Landry [19]	2012	USA	Pathology containing non-DTC
Lee [20]	2012	Korea	Pathology containing non-DTC
Lee [21]	2012	Korea	Data from this study were included in a later study [11]
Tae [22]	2012	Korea	Data from this study were included in a later study [13]
Tae [23]	2012	Korea	Pathology containing non-DTC
Aliyev [24]	2013	USA	Pathology containing non-DTC
Yi [25]	2013	Korea	Data from this study were included in a later study [16]
Lee [26]	2014	Korea	Data from this study were included in a later study [11]
Lee [27]	2014	Korea	Data from this study were included in a later study [11]
Song [28]	2014	Korea	Pathology containing non-DTC
Tae [29]	2014	Korea	Data from this study were included in a later study [13]
Song [30]	2015	Korea	Pathology containing non-DTC
Aroca [31]	2016	USA	Pathology containing non-DTC
Song [32]	2016	Korea	Pathology containing non-DTC
Sung [33]	2016	Korea	Data from this study were included in a later study [16]
Fregoli [34]	2017	Italy	Pathology containing non-DTC
Lee [35]	2021	Korea	Pathology containing non-DTC
Mattecucci [36]	2021	Italy	Pathology containing non-DTC
Rossi [37]	2022	Italy	Pathology containing non-DTC
Tzelnick [38]	2023	Israel	Pathology containing non-DTC

non-DTC, non-differentiated thyroid carcinoma.

### 3.3 Meta-analysis results

#### 3.3.1 Postoperative complications

**Fig 2:**
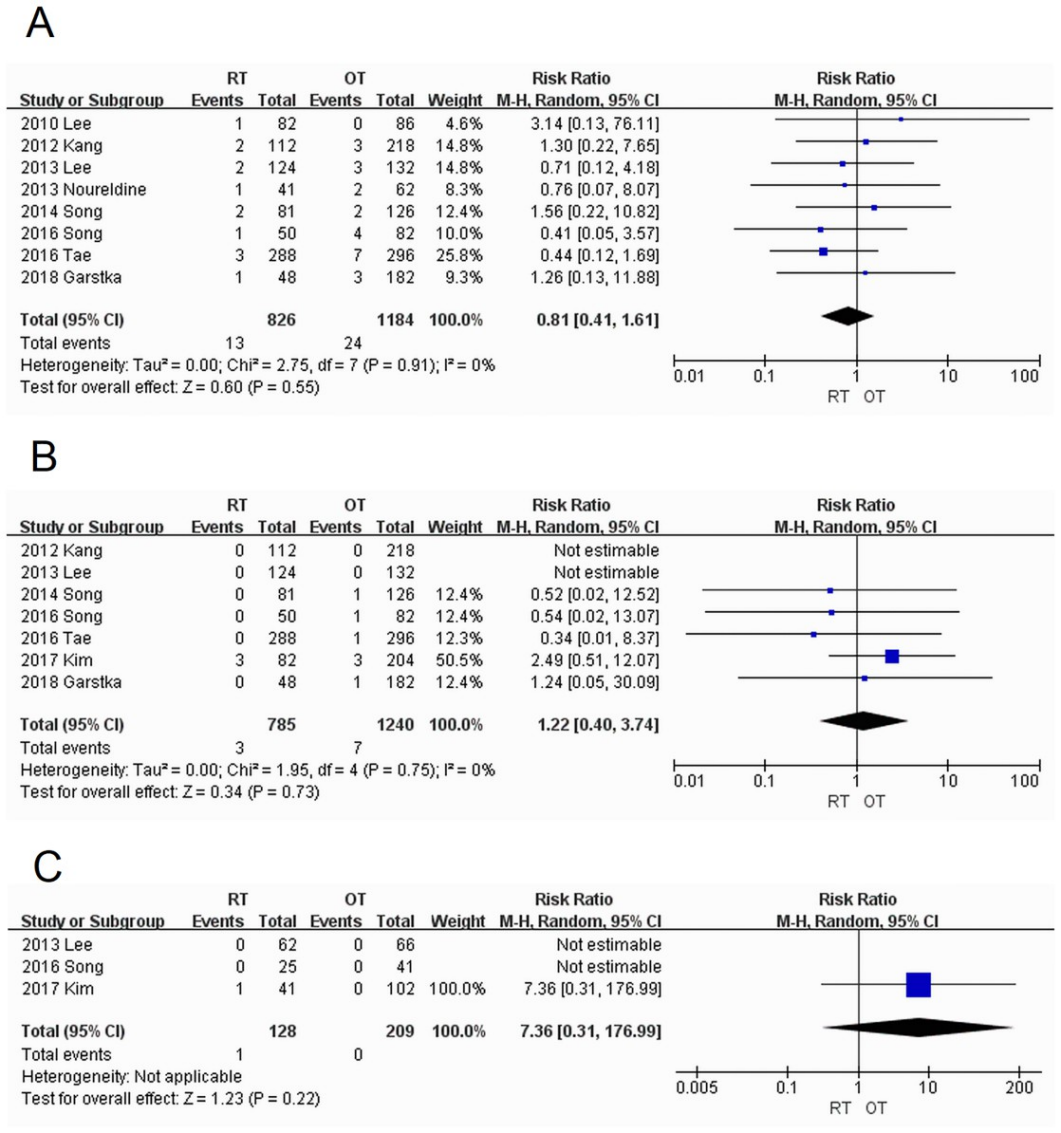
Forest plots displaying incidences of (A) transient recurrent laryngeal nerve injury; (B) permanent recurrent laryngeal nerve injury; (C) brachial plexus nerve injury. RT, robotic thyroidectomy; OT, open thyroidectomy; M-H, Mantel–Haenszel; CI, confidence interval.

*Transient recurrent laryngeal nerve injury*. A total of eight studies [5–8, 10, 12, 13, 15] were used to study temporary recurrent laryngeal nerve injury with low heterogeneity (I^2^ = 0%), wherein the injury proportion of 1.57% (13/826) in the RT group was slightly lower than that of 2.03% (24/1184) in the OT group, with no statistically significant difference (RR = 0.81; 95% CI:0.41–1.61; *P =* 0.55) (Fig 2A).

*Permanent recurrent laryngeal nerve injury*. A total of seven studies [6, 7, 10, 12–15] were included with low heterogeneity (I^2^ = 0%), and the difference was not statistically significant (RR = 1.22; 95% CI:0.40–3.74; *P* = 0.73). The proportion of injury in the RT group was 0.38% (3/785), which was slightly lower than that in the OT group (0.56%; 7/1240) (Fig 2B).

*Brachial plexus injury*. A total of three studies [7, 12, 14] were included, and only one study reported one case of brachial plexus injury in the RT group. The difference was not statistically significant (RR = 7.36; 95% CI:0.31–176.99; *P* = 0.22) (Fig 2C).

**Fig 3:**
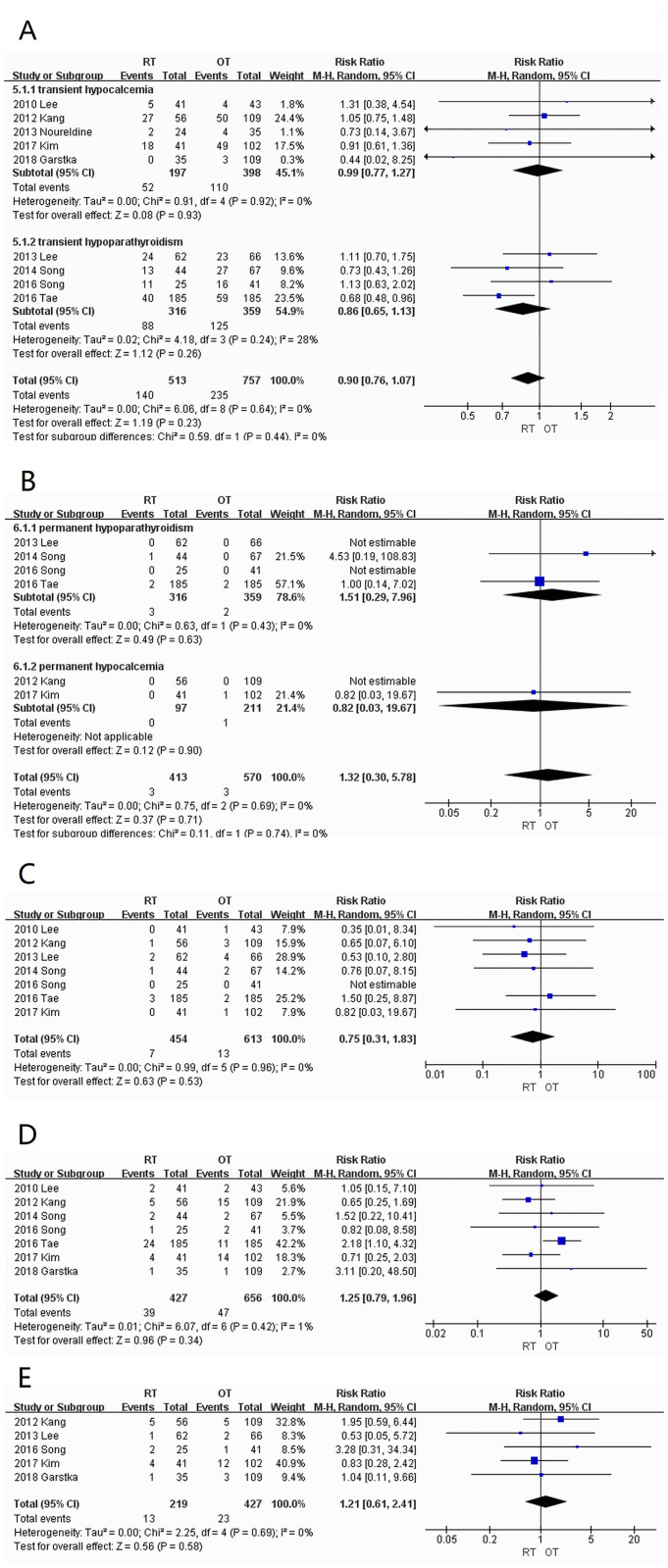
Forest plots displaying incidences of (A) transient hypocalcemia or hypoparathyroidism; (B) permanent hypocalcemia or hypoparathyroidism; (C) hematoma; (D) seroma; (E) lymphatic leakage RT, robotic thyroidectomy; OT, open thyroidectomy; M-H, Mantel–Haenszel; CI, confidence interval.

*Transient hypoparathyroidism or hypocalcemia*. Nine studies [5–8, 10, 12–15] were included with low heterogeneity (I^2^ = 0%). There was no statistically significant difference between the symptom incidences of 27.3% (140/513) in the RT group and 31.0% (235/757) in the OT group (RR = 0.90; 95% CI:0.76–1.07; *P* = 0.23) (Fig 3A).

*Permanent hypoparathyroidism or hypocalcemia*. Six studies [6, 7, 10, 12–14] were included with low heterogeneity (I^2^ = 0%). There was no significant difference (RR = 1.32; 95% CI:0.30–5.78; *P* = 0.71) between the RT (0.73%; 3/413) and OT (0.53%; 3/570) groups (Fig 3B).

*Hematoma*. Seven studies [5–7, 10, 12–14] were included with low heterogeneity (I^2^ = 0%). There was no significant difference (WMD = 0.75; 95% CI:0.31–1.83; *P* = 0.53) between the 1.5% (7/454) of hematomas in the RT group and 2.1% (13/613) in the OT group (Fig 3C).

*Seroma*. Seven studies [5, 6, 10, 12–15] were included with low heterogeneity (I^2^ = 1%). There was no significant difference (WMD = 1.25; 95% CI:0.79–1.96; *P* = 0.34) in the incidence of seroma in the RT (9.1%; 39/427) and OT (7.2%; 47/656) groups (Fig 3D).

*Lymphatic leakage*. Five studies [6, 7, 12, 14, 15] were included with low heterogeneity (I^2^ = 0%). There was no significant difference (WMD = 1.21; 95% CI:0.61–2.41; *P* = 0.58) in lymphatic leakage between the RT (5.9%; 13/219) and OT (5.4%; 23/427) groups (Fig 3E).

#### 3.3.2 Oncological outcomes

**Fig 4:**
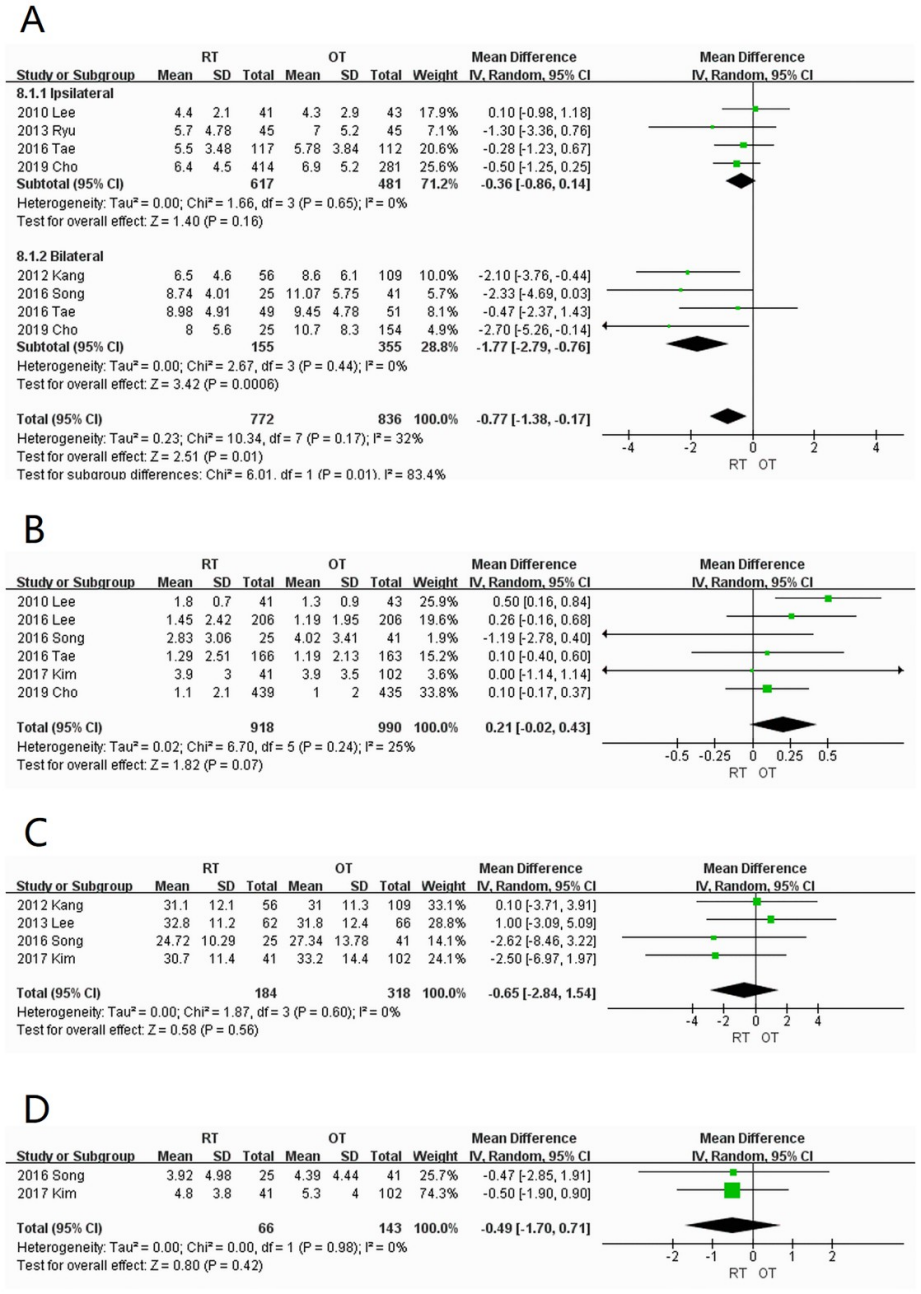
Forest plots displaying number of (A) retrieved central lymph nodes; (B) central lymph node metastases; (C) retrieved lymph nodes in the lateral cervical region; (D) metastatic lymph nodes retrieved from the lateral component RT, robotic thyroidectomy; OT, open thyroidectomy; SD, standard deviation; CI, confidence interval; IV, inverse variance.

*Number of retrieved central lymph nodes*. Six studies [5, 6, 9, 12, 13, 16] were included and divided into unilateral [5, 9, 12, 16] and bilateral [6, 12, 14, 16] subgroups according to the scope of dissection. Heterogeneity was low in both subgroups (I^2^ = 0%), but there was a significant difference in the number of retrieved central lymph nodes between the two groups. In the unilateral group, there was no significant difference between the RT and OT groups (WMD = -0.36; 95% CI: -0.86–0.14; *P* = 0.16). However, the number of retrieved central lymph nodes in the bilateral RT group was significantly lower than that in the OT group (WMD = -1.77; 95% CI: -2.79 –-0.76; *P* = 0.0006) (Fig 4A).

*Number of central lymph node metastases*. A total of six studies [5, 11–14, 16] were included with low heterogeneity (I^2^ = 25%). There was no significant difference between the two groups (WMD = 0.21; 95% CI: -0.02–0.43; *P* = 0.07) (Fig 4B).

*Number of retrieved lymph nodes in the lateral cervical region*. Four studies [6, 7, 12, 14] were included with low heterogeneity (I^2^ = 0%). There was no statistically significant difference between the RT and OT groups (WMD = -0.65; 95% CI: -2.84–1.54; *P* = 0.56) (Fig 4C).

*Number of metastatic lymph nodes in the lateral cervical region*. Two studies [12, 14] were included with low heterogeneity (I^2^ = 0%). There was no statistically significant difference between the RT and OT groups (WMD = -0.49; 95% CI: -1.70–0.71; *P* = 0.42) (Fig 4D).

#### 3.3.3 Operation-related events

**Fig 5:**
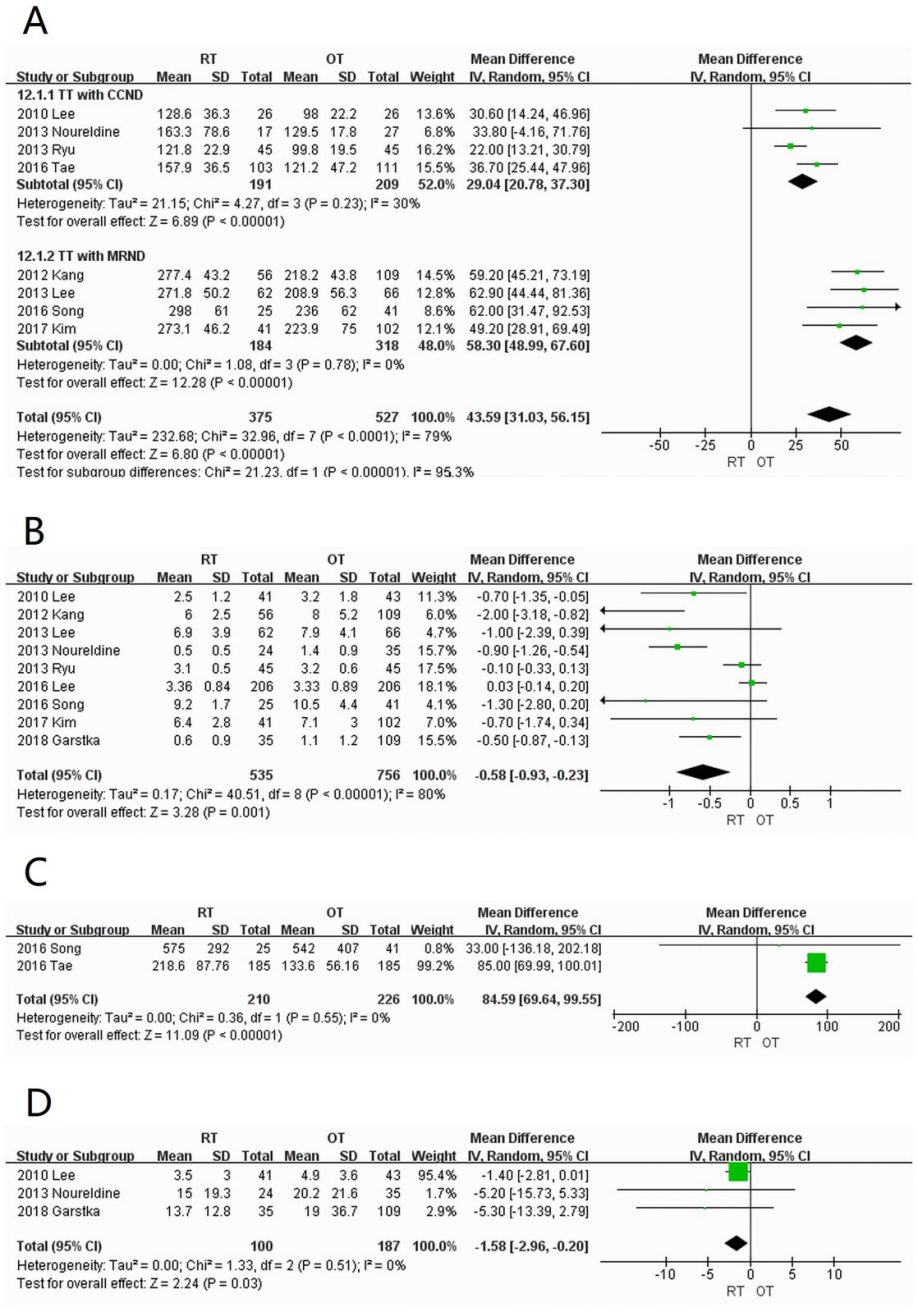
Forest plots displaying (A) operative time of total thyroidectomy; (B) length of hospitalization; (C) drain amount; (D) amount of intraoperative bleeding RT, robotic thyroidectomy; OT, open thyroidectomy; SD, standard deviation; CI, confidence interval; IV, inverse variance; TT, total thyroidectomy; CCND, central component lymph node dissection; MRND, modified radical lymph node dissection.

*Operation time of total thyroidectomy*. A total of eight studies [5–9, 12–14] were included for comparison of operation time. According to the scope of lymph node dissection, patients were divided into the central lymph node dissection [5, 8, 9, 13] and functional neck dissection [6, 7, 12, 14] subgroups. Heterogeneity was low in both groups at 30% and 0%, respectively. In the two subgroups, the operation time of the RT group was significantly longer than that of the OT group, and the differences in the central lymph node dissection subgroup (WMD = 29.04; 95% CI:20.78–37.30; *P* < 0.00001) and functional neck dissection subgroup (WMD = 58.30; 95% CI:48.99–67.60; *P* < 0.00001) were both statistically significant (Fig 5A).

*Length of hospitalization*. Nine studies [5–9, 11, 12, 14, 15] were included with high heterogeneity between the groups (I^2^ = 80%). The length of hospitalization in the RT group was significantly shorter than that in the OT group (WMD = -0.58; 95% CI: -0.93 –-0.23; *P* = 0.001) (Fig 5B).

*Volume of drainage*. Two studies [12, 13] were included with low heterogeneity (I^2^ = 0%). The RT group resulted in significantly more volume of drainage than the OT group (WMD = 84.59; 95% CI:69.64–99.55; *P* < 0.00001) (Fig 5C).

*Amount of intraoperative bleeding*. Three studies [5, 8, 15] were included with low heterogeneity (I^2^ = 0%). The results showed that the amount of blood loss in RT group was significantly less than that in OT group (WMD = -1.58; 95% CI: -2.96 –-0.20; *P* = 0.03) (Fig 5D).

#### 3.3.4 Postoperative events

**Fig 6:**
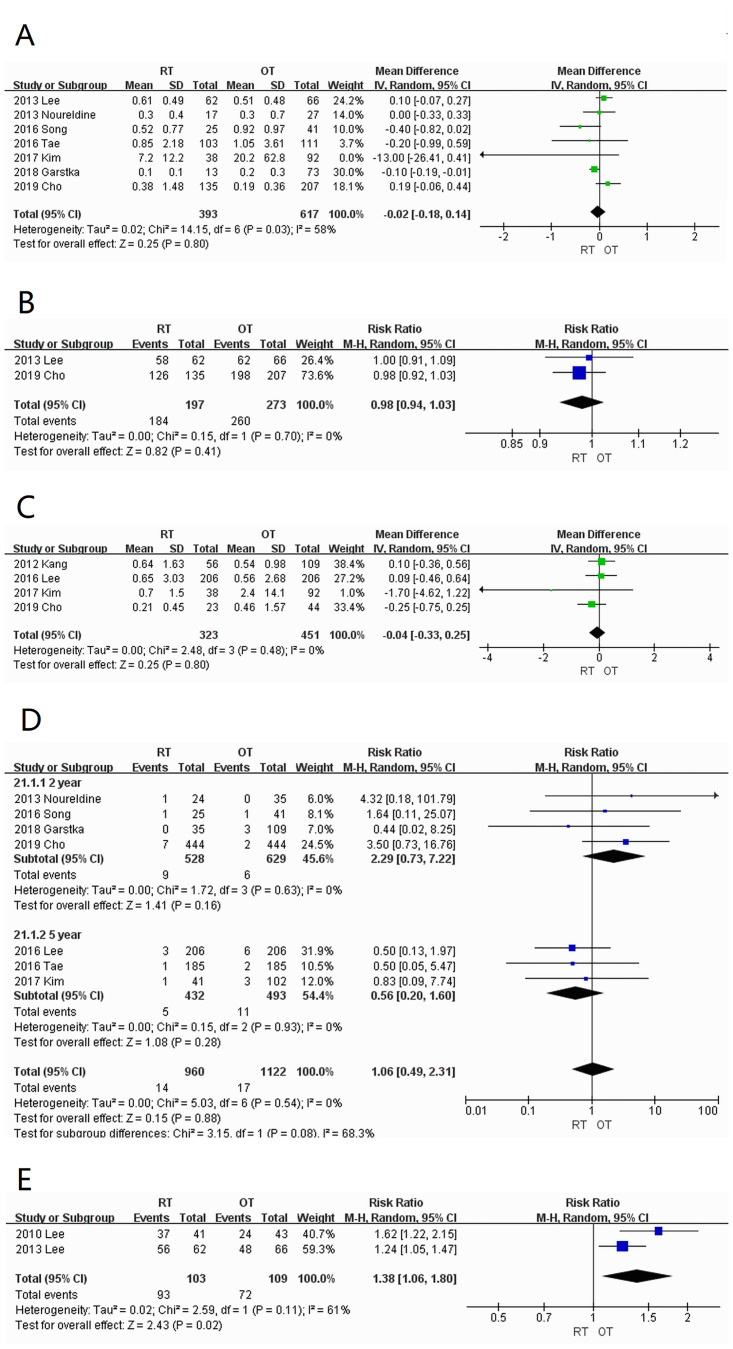
Forest plots displaying (A) sTg; (B) number of sTg<1ng/ml; (C) uTg; (D) recurrence rate; (E) cosmetic satisfaction RT, robotic thyroidectomy; OT, open thyroidectomy; SD, standard deviation; CI, confidence interval; IV, Inverse Variance.

*sTg (ng/ml) level*. Seven studies [7, 8, 12–16] were included with high heterogeneity (I^2^ = 58%). There was no significant difference (WMD = -0.02; 95% CI: -0.18–0.14; *P =* 0.80) in the sTg levels between the RT and OT groups (Fig 6A).

*sTg < 1ng/ml*. Only two studies [7, 16] had low heterogeneity (I^2^ = 0%). There was no significant difference (WMD = 0.98; 95% CI:0.94–1.03; *P* = 0.41) between the RT (93.4%; 184/197) and OT groups (95.2%; 260/273) (Fig 6B).

*uTg (ng/ml) level*. A total of four studies [6, 11, 14, 16] were included with low heterogeneity (I^2^ = 0%). There was no significant difference (WMD = -0.04; 95% CI: -0.33–0.25; *P* = 0.80) between the RT and OT groups (Fig 6C).

*Recurrence*. Seven studies [8, 11–16] were included. The recurrence rate was divided into two subgroups according to the follow-up periods of 2 and 5 years. The heterogeneity between the combined groups was low (I^2^ = 0%), and there was no significant difference (WMD = 1.06; 95% CI: 0.49–2.31; *P* = 0.88) in the recurrence rate of the RT (1.5%; 14/960) and OT (1.5%; 17/1122) groups (Fig 6D).

*Cosmetic satisfaction*. Two studies [5, 7] were included with high heterogeneity (I^2^ = 61%). The cosmetic satisfaction of the RT group was 90% (93/103), which was significantly (WMD = 1.38; 95% CI:1.06–1.80; *P* = 0.02) higher than that of the OT group (66%; 72/109) (Fig 6E).

## 4 Discussion

Generally speaking, DTC tends to occur in women between 40 and 50 years of age; however, the current trend is becoming younger [15]. Since the Da Vinci system was first used in thyroid surgery, different approaches have been proposed, which can be divided into two main approaches according to the operating direction: upward, bilateral axillo-breast approach, and unilateral axillary approach, as well as downward transoral and retroauricular approach [39]. Among these approaches, the transoral and retroauricular approaches are more difficult to operate because of the opposite surgical field due to the downward direction of the operation. In particular, the retroauricular approach has a small operating space and is difficult to perform due to the contralateral thyroid gland, resulting in contralateral incisions for total resection. In addition, the bilateral axillo-breast approach is difficult in patients with thoracic deformities and prominent clavicle protrusions. This method requires the injection of carbon dioxide to maintain the operating space, which may lead to hypercapnia, subcutaneous emphysema, and even gas embolism. At the same time, a large range of subcutaneous tissue separation is required because of the four incisions, which have a great impact on the sensation of the breast and chest wall. Compared to the bilateral axillo-breast approach, the unilateral axillary approach has fewer incisions and a smaller flap-free area [21].

Owing to these advantages, an increasing number of patients choose the unilateral axillary approach. Based on the experience of 5000 cases of unilateral axillary RT, Kim *et al*. [40] believed that this surgical procedure has the potential to reduce perioperative complications and achieve better surgical and oncological outcomes. To date, several early systematic reviews and meta-analyses of RT versus OT [2–4, 41–48] have been conducted, but the included studies were mixed with a variety of approaches, resulting in significant differences in the baseline characteristics of patients, high heterogeneity of results, and repeated inclusion of the same patient population, which affected the credibility of the results, which are shown in S2 Table. In this study, 12 included studies were strictly screened, and propensity score matching was used for four of them [11, 13, 14, 16]. It has been reported that propensity score matching can reduce selection bias, improve accuracy, and allow comparisons between different surgical procedures in nonrandomized retrospective studies [49, 50].

Compared with OT, the safety of unilateral axillary approach RT was the most important. The results of this meta-analysis showed that intraoperative blood loss in the RT group was significantly reduced compared to that in the OT group (*P* < 0.05). This is because the robotic system is equipped with 3D high-definition lens equipment, which can enlarge the surgical field of vision by 10–15 times, enabling the surgeon to identify fine vessels more clearly, enabling more precise hemostasis, and reducing the occurrence of bleeding.

However, the RT group had more postoperative drainage, and the difference was statistically significant compared to the OT group. Tae [13] believes that the range of RT-separated flaps is approximately three times that of OT, including the lateral neck and anterior chest wall. As the exudate would increase with the increase in surgical scope, this may be the reason for the significant increase in drainage after RT.

In addition, the total resection times in the two groups were significantly different. The patients were divided into two subgroups according to the scope of lymph node dissection because of the high heterogeneity, showing low heterogeneity in both groups. Regardless of the dissection method, the operation time in the RT group was significantly longer than that in the OT group. This may be related to the RT operation process, which includes three steps: creating the operation space, instrument docking, and the surgical operation. During the operation, the dense tissue in the neck region and lack of natural space made it necessary to conduct free dissection of the skin flap first, and the complex instrument docking adjustments and difficulty in the treatment of the contralateral thyroid lobe led to longer RT time. Lee *et al*. [7] found that the operation time of RT was similar to that of OT if the cavity construction and docking times were excluded, and most surgeons generally believed that the total operation time might be gradually shortened with the proficiency of surgical techniques [51, 52].

Another difference between the RT and OT groups was the length of the hospital stay. Some scholars believe that the discharge standards are that patients can walk freely after surgery, take drugs orally, tolerate pain, and have no surgically related complications [15]. However, Lee *et al*. [5] suggested that the drainage tube should be safely pulled out after surgery before discharge. This study showed that hospital stay in the RT group was shorter than that in the OT group, which may indicate that RT is safe and reliable. The high heterogeneity may be related to the different definitions of discharge criteria by surgeons; thus, the results should be treated with caution.

Except for the obvious differences mentioned above, there were no significant differences in most complications between the RT and OT groups (*P* > 0.05), including temporary and permanent recurrent laryngeal nerve injury, temporary and permanent hypoparathyroidism or hypocalcemia, brachial plexus nerve injury, hematoma, seroma, or lymphatic leak. Unlike manually controlled lenses in ET, the robotic system has a more stable field of vision, alleviates the surgeon’s sense of visual wobble and fatigue, and can better understand depth and distance. When dealing with the contralateral thyroid lobe, the camera could be rotated to ensure the surgical field of vision. For instance, if a deeper thyroid gland or forward protrusion of the trachea obstructs the operation, tilting the operating table contralateral upward by 10° to 15° can better expose the contralateral tracheoesophageal groove. Combined with the highly flexible manipulator, the surgeon is able to finely dissect the posterior thyroid peritoneum during the surgical operation, which facilitates the identification and protection of the recurrent laryngeal nerve and parathyroid glands, and thus may achieve the same results as open surgery [53].

In addition to safety, complications, and other variables, the completeness of total thyroidectomy has been of great concern and can be estimated postoperatively by ultrasound, uTg, sTg levels and whole-body scan iodine uptake levels. Since Tg can only be produced by normal thyroid or differentiated thyroid cancer cells, the clinical detection of Tg is simple, sensitive, and less invasive, and is considered a reliable indicator to measure the amount of residual tissue after total thyroidectomy. It has been proven that sTg less than 1 ng/ml after radioiodine therapy is a good marker for complete thyroidectomy [54]. Our study showed that the RT and OT groups had similar results with no statistical difference.

In addition, the number of cervical lymph node dissections is the main index for evaluating the thoroughness of surgery for DTC. Some studies have been conducted, but the high heterogeneity in most of them affects the reliability of the results [2, 4, 42]. In this study, heterogeneity was significantly reduced by dividing central lymph node dissection into unilateral and bilateral subgroups. The results showed no significant difference in the number of lymph nodes dissected from the unilateral central region between the two groups. However, when dissecting both sides, fewer lymph nodes were obtained in the RT group than in the OT group, and this difference was statistically significant. On one hand, obstruction of the trachea may be the reason. In this case, the field of vision and manipulation of instruments were limited, especially in the dissection of the posterior lymph nodes of the contralateral recurrent laryngeal nerve. However, some early published studies included in this study may be in the early exploratory stage of this operation, and the patients enrolled in RT belong to the early stage of the disease, resulting in a small number of dissected lymph nodes. In the future, lymph nodes may be removed more thoroughly as the operator becomes more proficient. Next, the analysis results showed that there were no significant differences in the number of central lymph node metastases, the number of retrieved lymph nodes in the lateral cervical region, and the number of metastases between RT and OT. Additionally, the number of retrieved lymph nodes in each group was significantly greater than the number of metastases, indicating that RT met the requirements of oncology safety. Finally, There was no statistically significant difference in the postoperative recurrence rate between the two groups. However, Heaton *et al*. showed that higher lymph node yield in central and lateral neck dissections is associated with lower rates of papillary thyroid cancer recurrence in the central and lateral neck [55]. Further studies are still needed.

Another noteworthy aspect was the cosmetic satisfaction rate after surgery for 3–6 months. The cosmetic satisfaction rate of patients in the RT group was significantly higher than that in the OT group. This is because the RT incision is in the axilla, which can be better hidden when the patient’s upper limb is in the natural state, while the OT neck incision is obvious, especially in patients with a scar constitution. This was the original intention for the design of endoscopic thyroid surgery.

With the rapid development of RT technology, the Food and Drug Administration approved the latest Da Vinci SP system, namely the single-hole Da Vinci Robot for clinical use in 2018, in which all joints and endoscopes were inserted through a single-arm chamber with two control joints. This type of robot can perform more complex fine operations in a smaller anatomical area, which may further improve the operation of RT and simplify tedious regulatory steps. Some scholars have conducted preliminary explorations [56].

This study systematically evaluated the efficacy of unilateral axillary approach robotic thyroid surgery compared with open thyroid surgery in the treatment of DTC but still had the following limitations: (1) The current research did not register on PROSPERO, although we strictly followed the steps of systematic review, there may still be bias [57]. (2) The 12 studies included in this meta-analysis were non-random or retrospective, and the criteria for temporary/permanent recurrent laryngeal nerve injury, temporary/permanent hypoparathyroidism, and hypocalcemia in each study may be different, although some studies have adopted the propensity score matching method, there may still exist bias. (3) The studies included originate all from the US and Korea, and some of these publications are authored by the same surgical teams, thus this meta-analysis may primarily reflect the experiences of a limited number of experts, potentially limiting the generalizability of the study results. (4) Most of the follow-up periods included in the literature were short. Since DTC is a relatively indolent malignant tumor, a longer follow-up period may be needed to evaluate the oncological safety of surgery.

## 5 Conclusion

Unilateral axillary robotic thyroid surgery may achieve results similar to those of traditional open surgery. Although the RT group had a longer total thyroidectomy time and more postoperative drainage, it showed shorter hospital stays, less intraoperative bleeding, less retrieved central lymph nodes, and higher cosmetic satisfaction, wherein all the differences were statistically significant (*P* < 0.05). To better understand RT, further validation in large prospective randomized controlled trials and long-term follow-up is required.

## Supporting information

S1 Table Systematic search strategy (PICOS strategy). (DOCX)

S2 Table Up-to-date meta-analyses (including robotic trans-axillary approach). (DOCX)

S1 Dataset Original data extraction table. (ZIP)

S1 Checklist PRISMA 2020 checklist. (DOCX)
