# Supplementary material for: A meta-analysis of unilateral axillary approach for robotic surgery compared with open surgery for differentiated thyroid carcinoma
Source: PLoS One. 2024 Apr 11;19(4):e0298153. doi: 10.1371/journal.pone.0298153 (PMC11008900; doi:10.1371/journal.pone.0298153)
Supplement: S1 Table — (DOCX) [file pone.0298153.s001.docx]

S1 Table. Systematic search strategy (PICOS strategy)

| Search strategy |  |
| --- | --- |
| Population | #1. "thyroidectomy"[MeSH Terms] OR "thyroidectom*"[Title/Abstract] OR "thyreoidectom*"[Title/Abstract] OR "thyroid surger*"[Title/Abstract] OR "thyroid operation*"[Title/Abstract] |
|  | #2. "neoplasms"[MeSH Terms] OR "neoplas*"[Title/Abstract] OR "tumor*"[Title/Abstract] OR "cancer*"[Title/Abstract] OR "malignanc*"[Title/Abstract] OR "malignant neoplasm*"[Title/Abstract] |
| Intervention | #3. "robotics"[MeSH Terms] OR "robot*"[Title/Abstract] OR "remote operation*"[Title/Abstract] OR "Telerobotics"[Title/Abstract] OR "soft robotic*"[Title/Abstract] OR "robot surger*"[Title/Abstract] OR "robot assisted surger*"[Title/Abstract] OR "robot enhanced procedure*"[Title/Abstract] OR "robot enhanced surger*"[Title/Abstract] OR "robotic assisted surger*"[Title/Abstract] OR "robotic surgical procedure*"[Title/Abstract] |
| Comparisons | #4. "open"[Title/Abstract] OR "conventional"[Title/Abstract] |
| Outcomes | No restriction |
| Study design | No restriction |
| Search  combination | #1 AND #2 AND #3 AND #4 |
| Language | English |
| Electronic  databases | PubMed, Embase, Cochrane Library, and Web of Science |
