# Supplementary material for: A meta-analysis of unilateral axillary approach for robotic surgery compared with open surgery for differentiated thyroid carcinoma
Source: PLoS One. 2024 Apr 11;19(4):e0298153. doi: 10.1371/journal.pone.0298153 (PMC11008900; doi:10.1371/journal.pone.0298153)
Supplement: S2 Table — (DOCX) [file pone.0298153.s002.docx]

**S2 Table. Up-to-date meta-analyses (including robotic trans-axillary approach)**

| Authoryear | No.of studies | No.of patients | outcomes | comparison arms |
| --- | --- | --- | --- | --- |
| Lang 2014 | 10 | 2205 | Number of central lymph nodes (CLNs) retrieved during central neck dissection (CND), preablation stimulated thyroglobulin (sTg) level, radioiodine uptake on post therapy scan, and locoregional recurrence (LRR) | RT and OT |
| Pan 2017 | 23 | 5200 | Operation time, Blood loss and total drain amount, Length of hospital stay, Metastatic and retrieved lymph node, Postoperative Tg level, pain score, cosmetic,Satisfaction, Hypocalcemia and hypoparathyroidism, RLN palsy and other complications, postoperative RAI ablation rate Recurrence rate | RT and OT |
| Kandil 2015 | 18 | 4878 | Total Operative Time, Length of Hospital Stay, Postoperative Complications, Postoperative Pain, Thyroglobulin Level, Intraoperative Blood Loss | RT,ET and OT |
| Shen 2014 | 9 | 1615 | postoperative complications,number of retrieved lymph nodes, operative time, postoperative hospital stay, and cosmetic satisfaction | RT and OT |
| Wang 2015 | 12 | 2513 | operative time, number of retrieved central lymph nodes, transient RLN palsy, permanent RLN palsy, transient hypocalcaemia, permanent hypocalcaemia, chyle leakage, post-operative hospital stay, post-operative suppressed serum thyroglobulin levels, post-operative TSH stimulated serum thyroglobulin levels | RT and OT |
| Jackson 2012 | 9 | 2881 | Operative time, Length of hospital stay, Postoperative pain, Cosmetic satisfaction, Postoperative TG levels | RT,ET and OT |
| Sun 2014 | 11 | 1931 | Operative time, Hospital length of stay, hematoma, seroma, recurrent laryngeal nerve (RLN) injury, hypocalcemia, chyle leak, brachial plexus injury, Voice, Swallowing, Cosmetic satisfaction, Pain, Paresthesia | RT and OT |
| Son 2015 | 14 | 3136 | Mortality, Tracheal injury, conversion rate, ransient and permanent hypocalcemia, Operation times, Blood Loss, Cosmetic Satisfaction After 3 Months, Swallowing, Retrieved Lymph Nodes, Postoperative Thyroglobulin Level, | RT and OT |
| Sukato 2018 | 11 | 655 | Operative Time and Length of Stay, chyle leak, hematoma, Horner’s syndrome, marginal man dibular nerve weakness, seroma, wound infection, and surgical wound drainage, Total Lateral Nodal Yield, Pathological Yield,and Recurrence Rate, Cosmesis, | RT and OT |
| Liu 2020 | 59 | 163984 | Operative duration, Hospitalization, Intraoperative blood loss, Total drain amount and duration, Recurrent laryngeal nerve (RLN) palsy, Hypoparathyroidism or hypocalcemia, Bleeding and hematoma, seroma, and chyle leakage, rachial plexus injury, vagus nerve injury, earlobe numbness, tracheal injury, infection, Number of retrieved lymph nodes, Recurrence rate, Postoperative thyroglobulin (Tg) level, Postoperative pain and paresthesia, Voice assessment, Cosmetic satisfaction | RT and OT |
