# Supplementary material for: A meta-analysis of unilateral axillary approach for robotic surgery compared with open surgery for differentiated thyroid carcinoma
Source: PLoS One. 2024 Apr 11;19(4):e0298153. doi: 10.1371/journal.pone.0298153 (PMC11008900; doi:10.1371/journal.pone.0298153)

**Title:** **Transaxillary robotic modified radical neck dissection: a 5-year assessment of operative and oncologic outcomes**

**Study design**: Cohort study with propensity score matching Quality score: 9

**Author**: Min Jhi Kim

**Year**:2017

**Address**: Korea Yonsei University Cancer Center

**Surgeon**: Woong Youn Chung

**Surgery approach**: unilateral axillary approach

**Surgery time**:2007.09-2010.02

**Surgery extent**: Total thyroidectomy(TT) with central compartment neck dissection(CCND) and modified radical neck dissections (MRND)

**Inclusion Criteria**: PTC patients with clinically palpable lateral neck lymph nodes (LNs) or suspicious-appearing lateral LNs on ultrasonography (US) underwent fine-needle aspiration biopsy (FNAB). Lateral LN metastases were evaluated preoperatively by US-guided FNAB or by measuring thyroglobulin (Tg) concentrations in FNAB wash-out fluid.

**Exclusion criteria**: (1) a history of previous neck surgery or irradiation; (2) unrelated pathologic conditions of the neck or shoulder; (3) suspected tumor invasion of an adjacent organ, such as the recurrent laryngeal nerve (RLN), esophagus, or trachea; (4) suspected perinodal infiltration of adjacent structures, such as the internal jugular vein or major nerves by lateral metastatic LNs; (5) known recurrent disease at the time of evaluation; or (6) distant metastasis.

**Permanent recurrent laryngeal nerve injury**: more than 6 months

**Permanent hypoparathyroidism/hypocalcemia**: more than 6 months

**Follow-up**:66.6 months


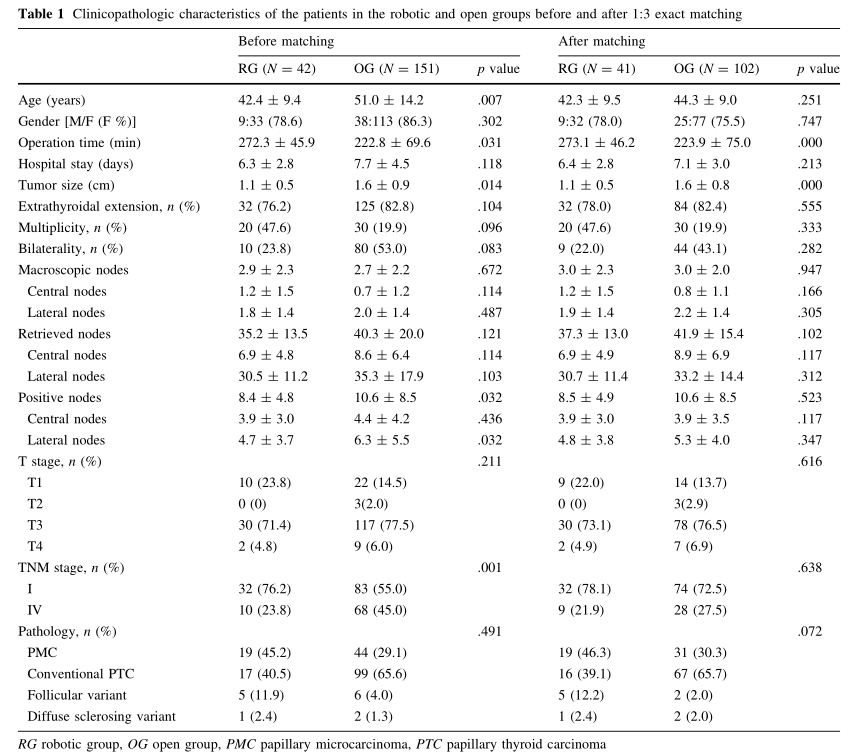


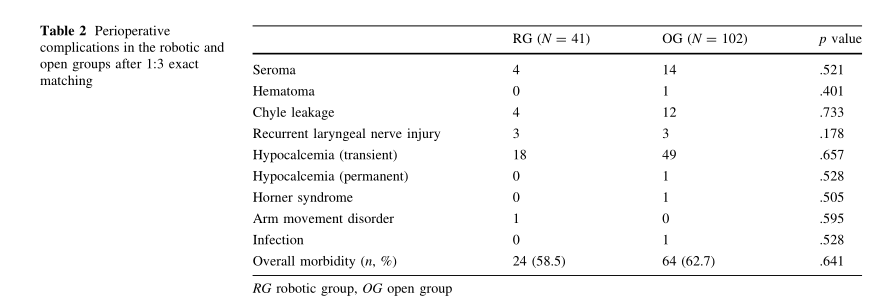


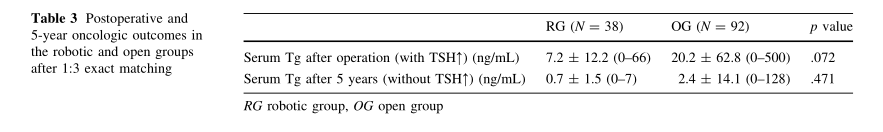


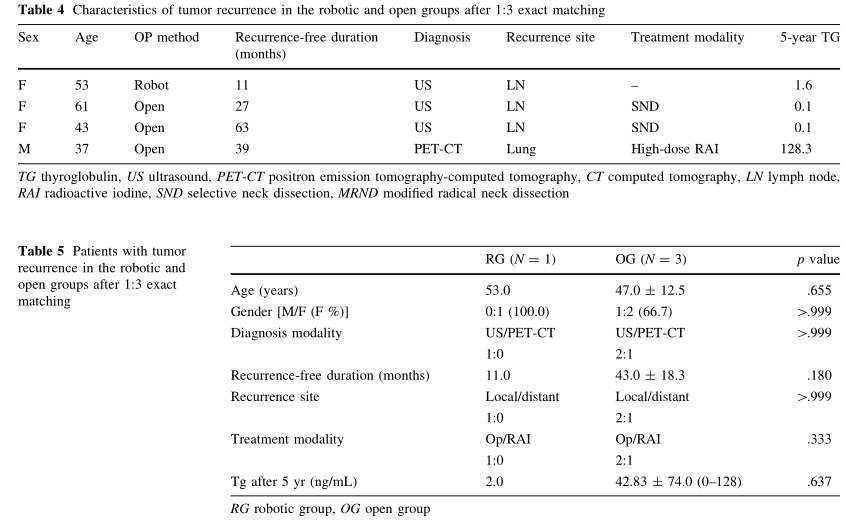

Supplement: S1 Dataset — (ZIP) [file pone.0298153.s003.zip › Data Set/10[14].docx]
