# Supplementary material for: A meta-analysis of unilateral axillary approach for robotic surgery compared with open surgery for differentiated thyroid carcinoma
Source: PLoS One. 2024 Apr 11;19(4):e0298153. doi: 10.1371/journal.pone.0298153 (PMC11008900; doi:10.1371/journal.pone.0298153)

**Title:** **Well-differentiated thyroid cancer and robotic transaxillary surgery at a North American institution**

**Study design**: Cohort study Quality score: 8

**Author**: Meghan Garstka

**Year**:2018

**Address**: USA Tulane University School of Medicine

**Surgeon**: Emad Kandil

**Surgery approach**: unilateral axillary approach

**Surgery time**:2015.01-2017.06

**Surgery extent**: Total thyroidectomy(TT) or lobectomy with central compartment neck dissection(CCND) and modified radical neck dissections (MRND)

**Inclusion Criteria**: All patients who underwent robotic-assisted or conventional open cervical thyroid resection with or without central neck dissection and MRND for well-differentiated thyroid cancer.

**Exclusion criteria**: Parathyroid surgeries and surgeries for benign thyroid diseases were excluded.

**Permanent recurrent laryngeal nerve injury**: more than 6 months

**Permanent hypoparathyroidism/hypocalcemia**: more than 6 months

**Follow-up**:7.3±6.1 months, 9.9±8.8 months


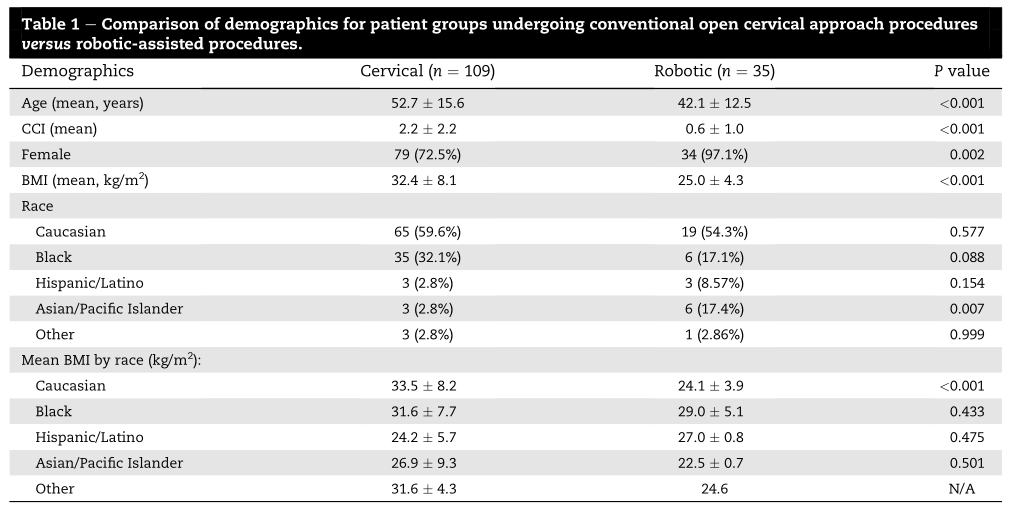


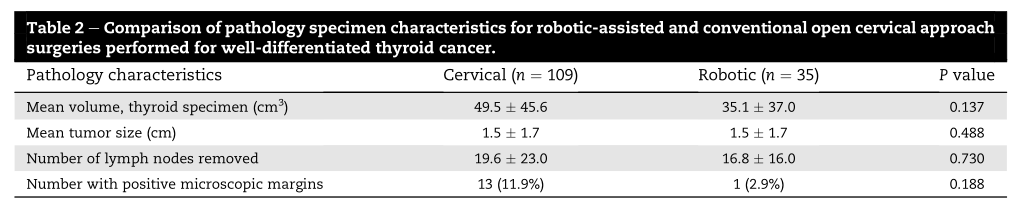


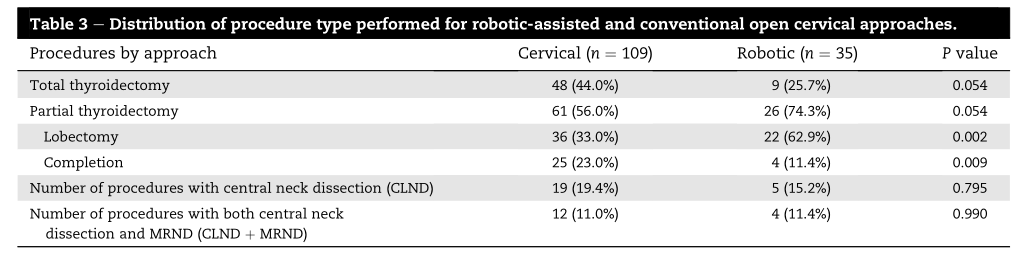


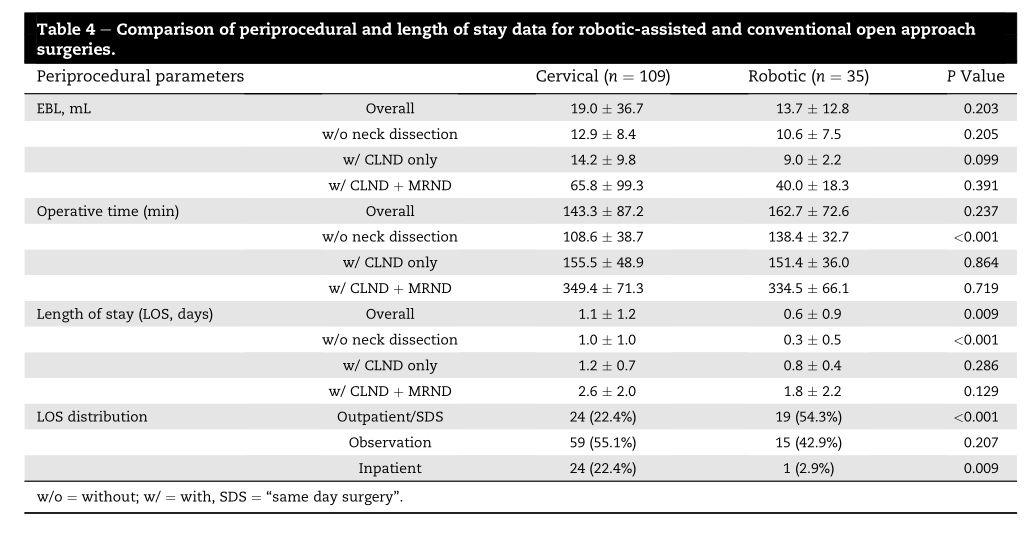


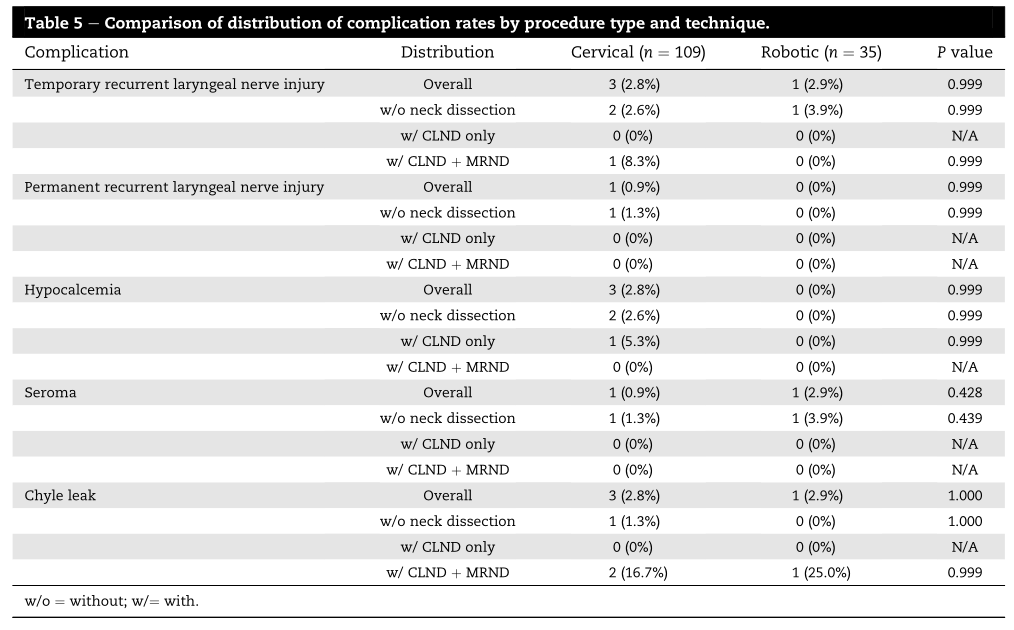


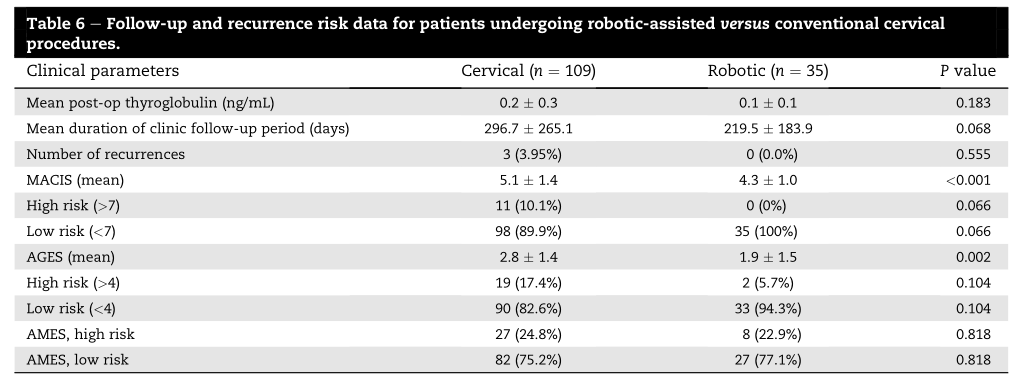

Supplement: S1 Dataset — (ZIP) [file pone.0298153.s003.zip › Data Set/11[15].docx]
