# Supplementary material for: A meta-analysis of unilateral axillary approach for robotic surgery compared with open surgery for differentiated thyroid carcinoma
Source: PLoS One. 2024 Apr 11;19(4):e0298153. doi: 10.1371/journal.pone.0298153 (PMC11008900; doi:10.1371/journal.pone.0298153)

**Title:** **Comparison of short-term oncologic outcome of robotic thyroid surgery using dynamic risk stratification: A propensity score–matched comparison study**

**Study design:** Cohort study with propensity score matching Quality score:9

**Author**: Jae Won Cho

**Year**:2019

**Address**: Korea Asan Medical Center

**Surgeon**: Jong Ho Yoon

**Surgery approach**: unilateral axillary approach

**Surgery time**:2008.12-2014.04

**Surgery extent**: Total thyroidectomy(TT) or lobectomy with central compartment neck dissection(CCND)

**Inclusion Criteria**: Before 2014 ,(1) PTC with a maximum diameter of ≤ 2 cm and (2) minimal invasion to the anterior thyroid capsule and strap muscle.Since 2014, the inclusion criteria have been expanded to include a primary tumor size up to 4 cm.

**Exclusion criteria**: definite posterior capsular invasion particularly adjacent to the tracheoesophageal groove, lateral LN metastasis, and distant metastasis at presentation.

**Permanent recurrent laryngeal nerve injury**: unclear

**Permanent hypoparathyroidism/hypocalcemia**: unclear

**Follow-up**:28-99 months


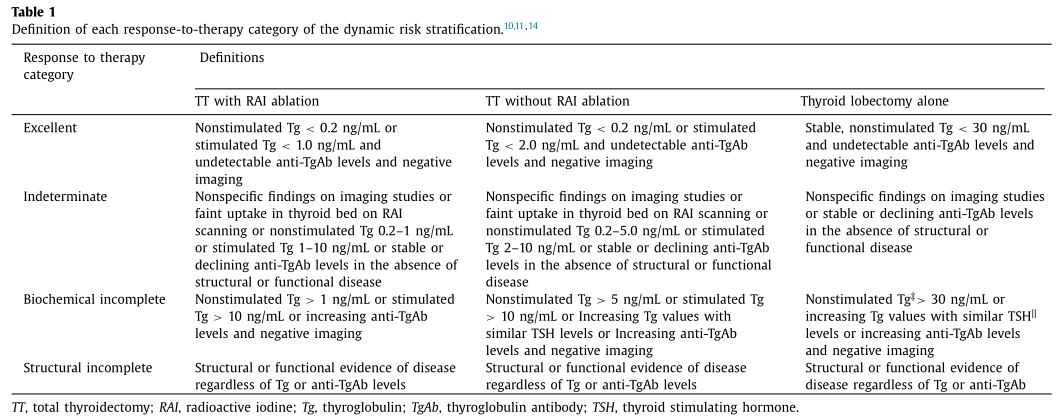


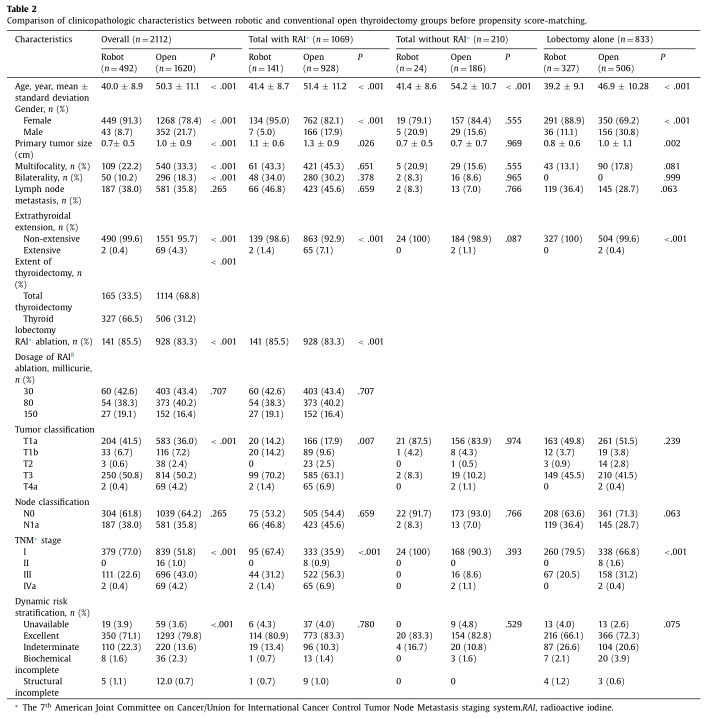


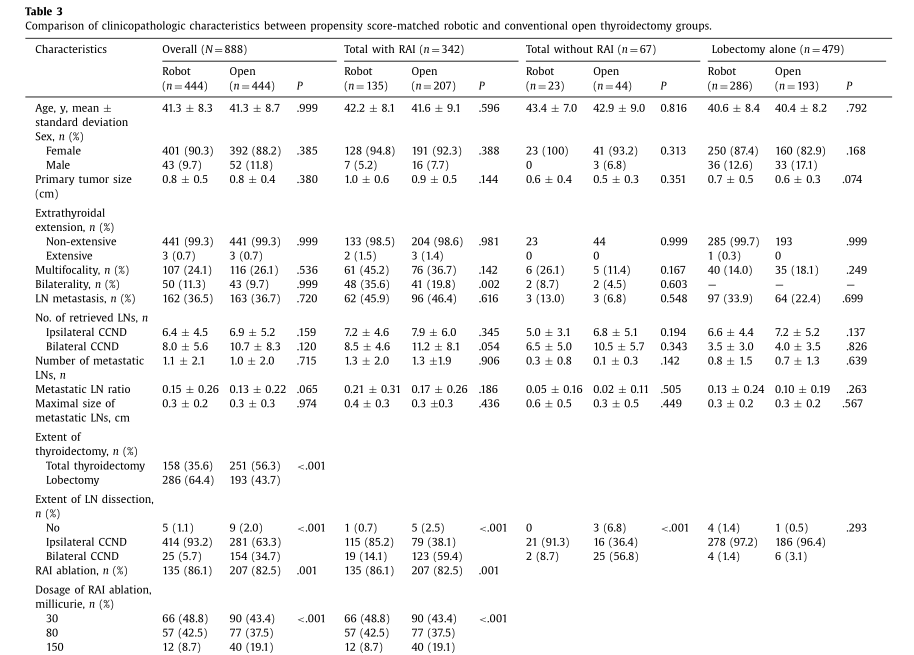

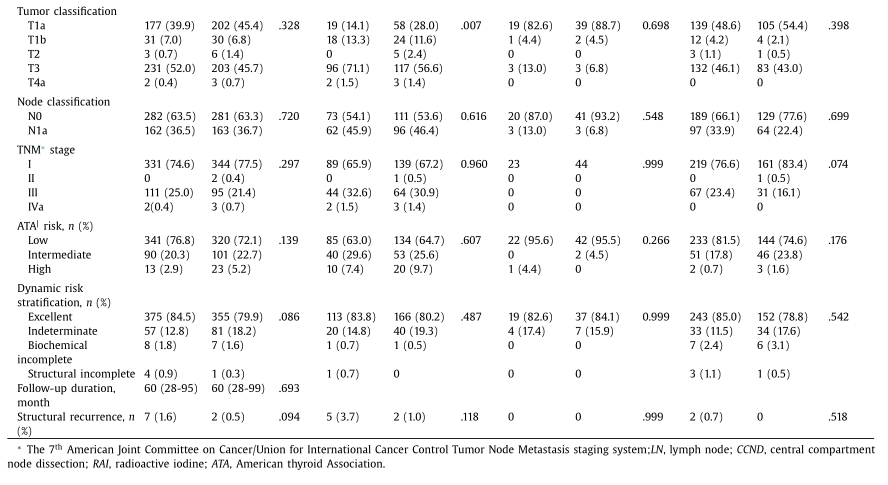


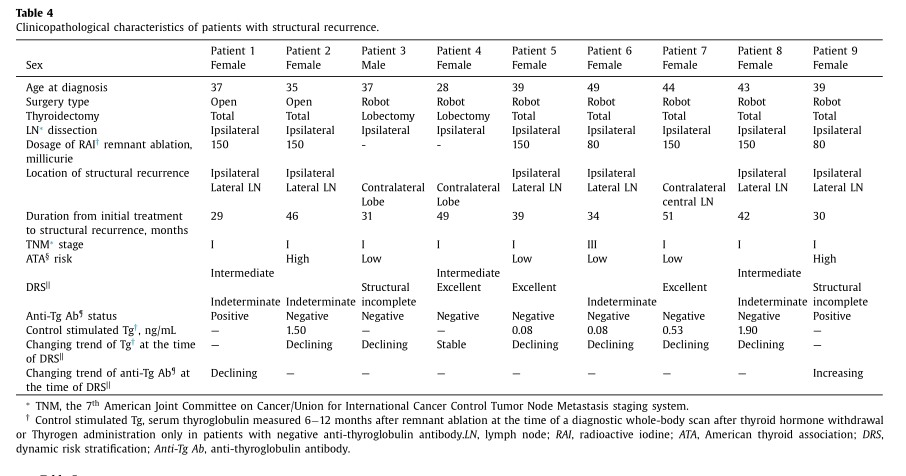


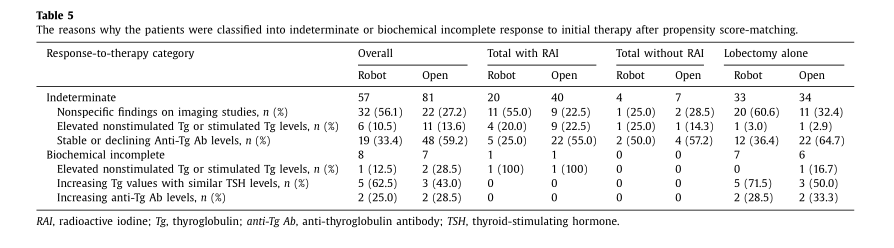


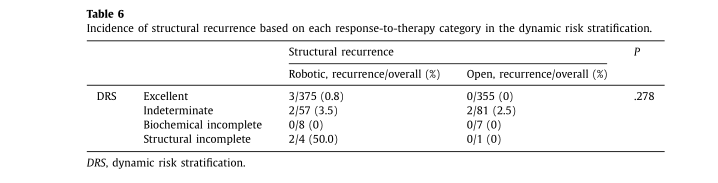


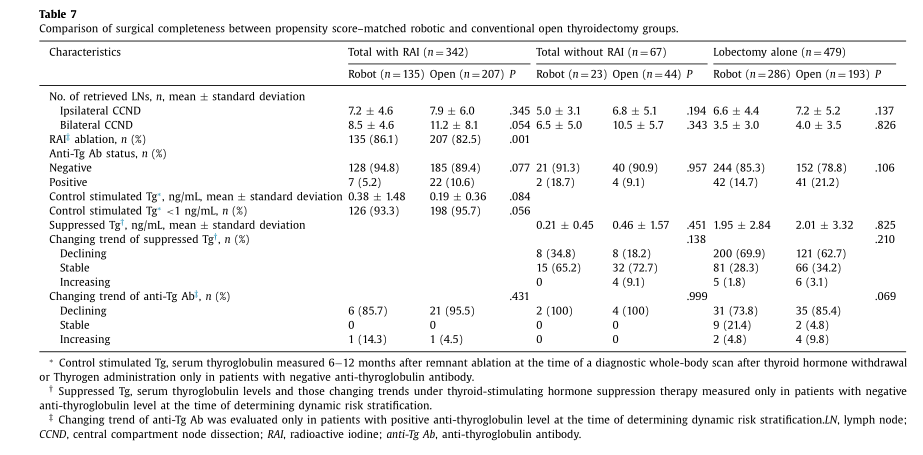

Supplement: S1 Dataset — (ZIP) [file pone.0298153.s003.zip › Data Set/12[16].docx]
