# Supplementary material for: A meta-analysis of unilateral axillary approach for robotic surgery compared with open surgery for differentiated thyroid carcinoma
Source: PLoS One. 2024 Apr 11;19(4):e0298153. doi: 10.1371/journal.pone.0298153 (PMC11008900; doi:10.1371/journal.pone.0298153)

**Title:Differences in postoperative outcomes, function, and cosmesis:open versus robotic thyroidectomy**

**Study design**: non-randomized controlled study(NRS) Quality score:17

**Author**: Jandee Lee

**Year**:2010

**Address**: Korea Yonsei University College of Medicine

**Surgeon**: Jandee Lee

**Surgery approach**: unilateral axillary approach

**Surgery time**:2009.04-2010

**Surgery extent**: Total thyroidectomy(TT) or subtotal thyroidectomy(STT) with ipsilateral central Compartment node dissection (CCND).

**Inclusion Criteria**: Patients had a minimally invasive follicular thyroid carcinoma 4 cm or less in diameter or a papillary thyroid carcinoma 2 cm or less in diameter.

**Exclusion criteria**: Patients with previous neck operations; age younger than 21 or older than 65 years; prior vocal fold paralysis or a history of voice or laryngeal disease requiring therapy; a malignancy with definite extrathyroid invasion, multiple lateral neck node metastases, perinodal infiltration at a metastatic lymph node or distant metastasis; or a lesion located in the thyroid dorsal area (especially adjacent to the tracheoesophageal groove) caused by possible injury to the trachea, esophagus, or recurrent laryngeal nerve (RLN).

**Permanent recurrent laryngeal nerve injury**: more than 6 months

**Permanent hypoparathyroidism/hypocalcemia**: unclear

**Follow-up**:3 months


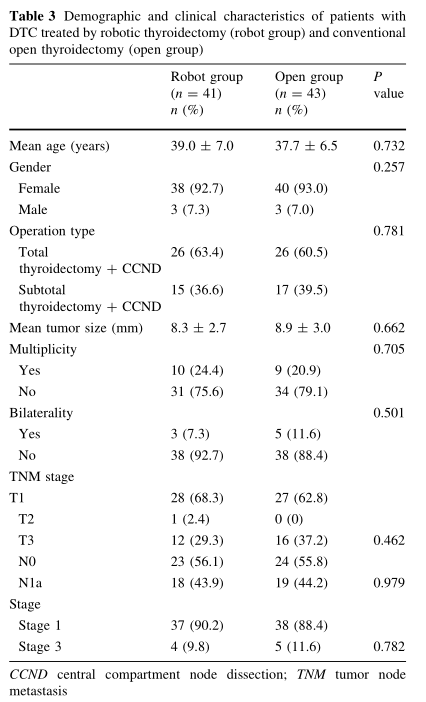


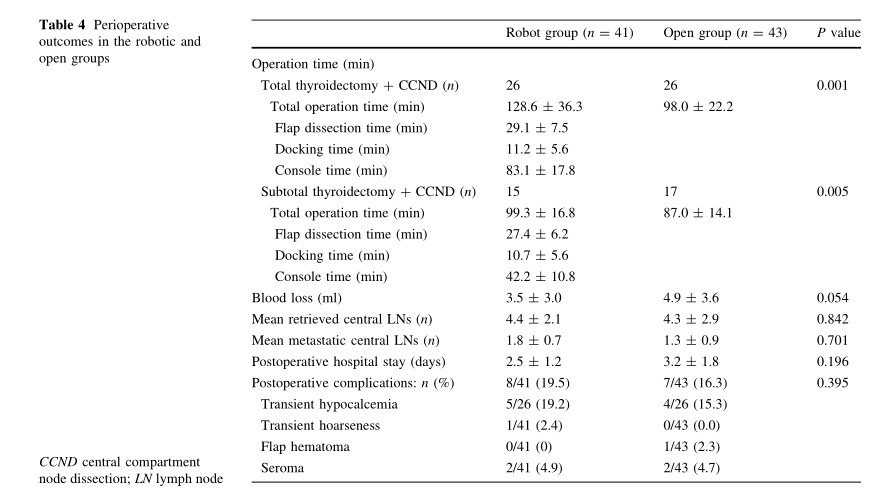


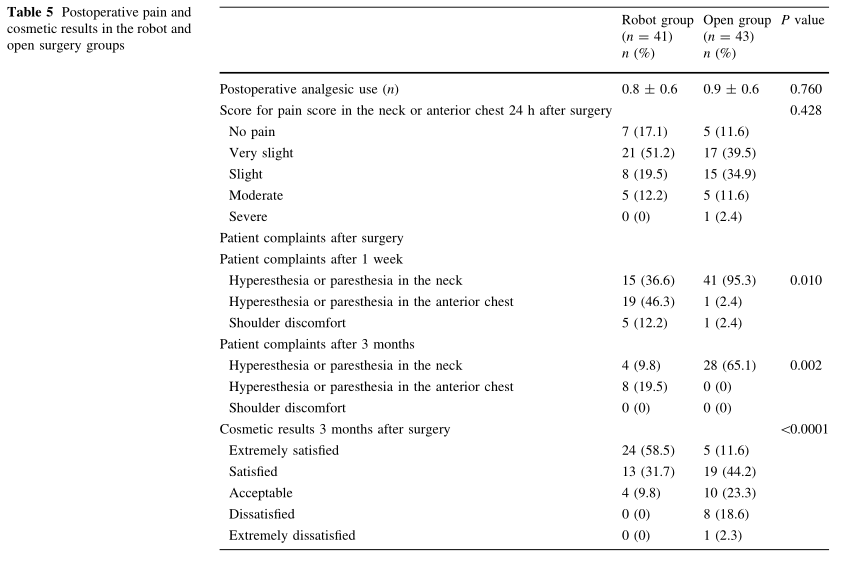


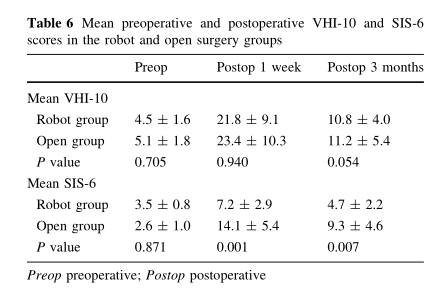

Supplement: S1 Dataset — (ZIP) [file pone.0298153.s003.zip › Data Set/1[5].docx]
