# Supplementary material for: A meta-analysis of unilateral axillary approach for robotic surgery compared with open surgery for differentiated thyroid carcinoma
Source: PLoS One. 2024 Apr 11;19(4):e0298153. doi: 10.1371/journal.pone.0298153 (PMC11008900; doi:10.1371/journal.pone.0298153)

**Title:** **A comparative study of the surgical outcomes of robotic and conventional open modified radical neck dissection for papillary thyroid carcinoma with lateral neck node metastasis**

**Study design**: Cohort study Quality score: 7

**Author**: Sang-Wook Kang

**Year**:2012

**Address**: Korea Yonsei University College of Medicine

**Surgeon**: Woong Youn Chung

**Surgery approach**: unilateral axillary approach

**Surgery time**:2009.01-2010.05

**Surgery extent**: Total thyroidectomy(TT) with central compartment neck dissection(CCND) and modified radical neck dissections (MRND)

**Inclusion Criteria**: (1) well differentiated thyroid carcinoma with clinical LNM (cases with one or two minimal metastatic LNs in the lateral neck), (2) tumor size≤4 cm, and (3) minimal invasion into the anterior thyroid capsule and strap muscle.

**Exclusion criteria**: (1) definite tumor invasion to an adjacent organ [recurrent laryngeal nerve (RLN), esophagus, or trachea] and (2) multilevel LN metastases in the lateral neck or perinodal infiltration at a metastatic lymph node.

**Permanent recurrent laryngeal nerve injury**: unclear

**Permanent hypoparathyroidism/hypocalcemia**: unclear

**Follow-up**:12 months


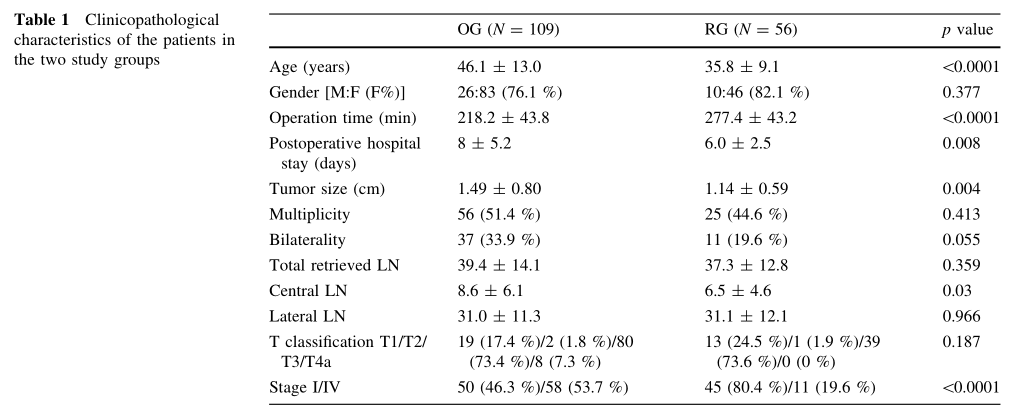


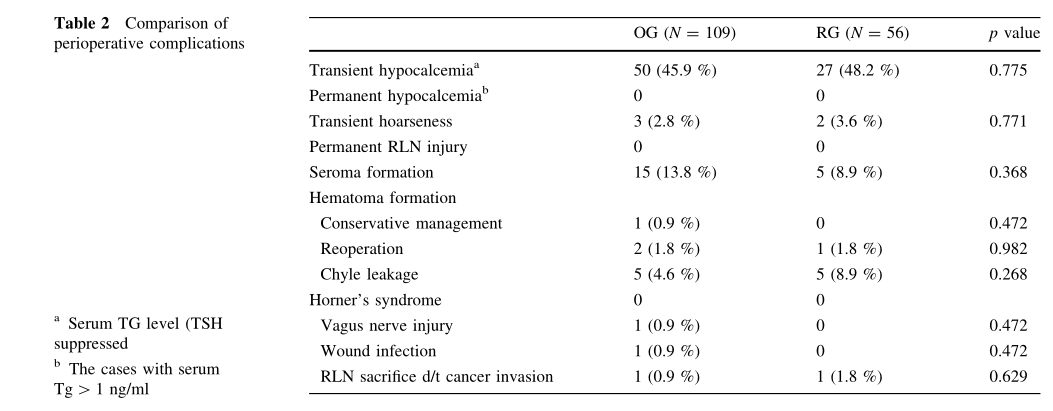


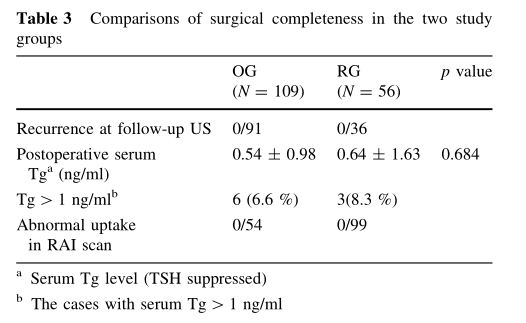

Supplement: S1 Dataset — (ZIP) [file pone.0298153.s003.zip › Data Set/2[6].docx]
