# Supplementary material for: A meta-analysis of unilateral axillary approach for robotic surgery compared with open surgery for differentiated thyroid carcinoma
Source: PLoS One. 2024 Apr 11;19(4):e0298153. doi: 10.1371/journal.pone.0298153 (PMC11008900; doi:10.1371/journal.pone.0298153)

**Title:** **Comparative Analysis of Oncological Outcomes and Quality of Life After Robotic versus Conventional Open Thyroidectomy With Modified Radical Neck Dissection in Patients With Papillary Thyroid Carcinoma and Lateral Neck Node Metastases**

**Study design:** non-randomized controlled study(NRS) Quality score:21

**Author**: Jandee Lee

**Year**:2013

**Address**: Korea Severance Hospital

**Surgeon**: Woong Youn Chung

**Surgery approach**: unilateral axillary approach

**Surgery time**:2010.06-2011.07

**Surgery extent**: Total thyroidectomy(TT) with central compartment neck dissection(CCND) and modified radical neck dissections (MRND)

**Inclusion Criteria**: patients had clinically palpable lateral neck LNs or lateral LNs with a suspicious appearance on ultrasonography (US), as shown by preoperative staging US, and underwent fine-needle aspiration cytology (FNAC). Lateral LN metastases were evaluated preoperatively by US-guided FNAC and by Tg concentrations in FNAC wash out fluid.

**Exclusion criteria**: 1) a history of previous neck surgery or irradiation; 2) unrelated pathologic conditions of the neck or shoulder; 3) known recurrent disease at the time of evaluation; 4) suspicious tumor invasion of an adjacent organ, such as the recurrent laryngeal nerve(RLN), esophagus,ortrachea;5) suspicious perinodal infiltration to adjacent structures, such as the internal jugular vein or major nerves for lateral metastatic LNs; or 6) distant metastases.

**Permanent recurrent laryngeal nerve injury**: more than 6 months

**Permanent hypoparathyroidism/hypocalcemia**: more than 6 months

**Follow-up**:12 months


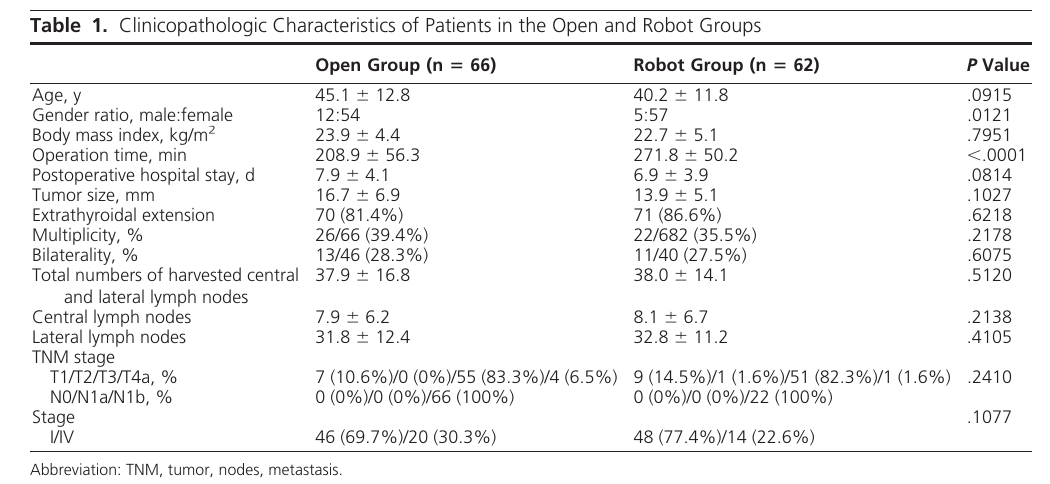


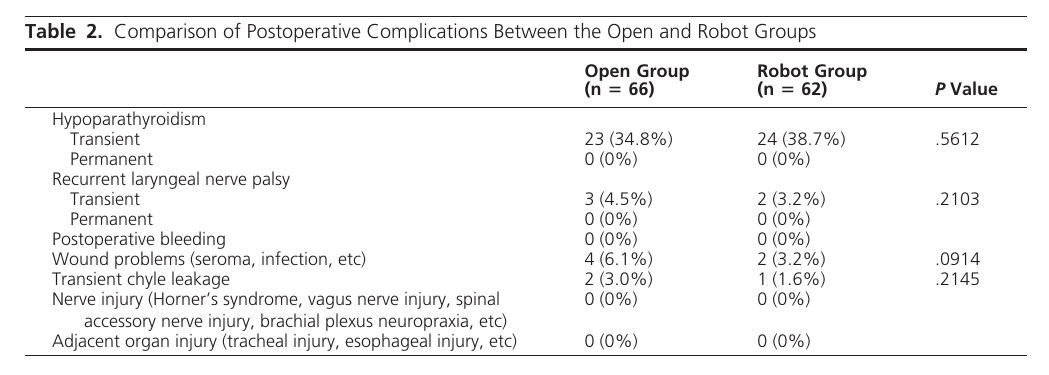


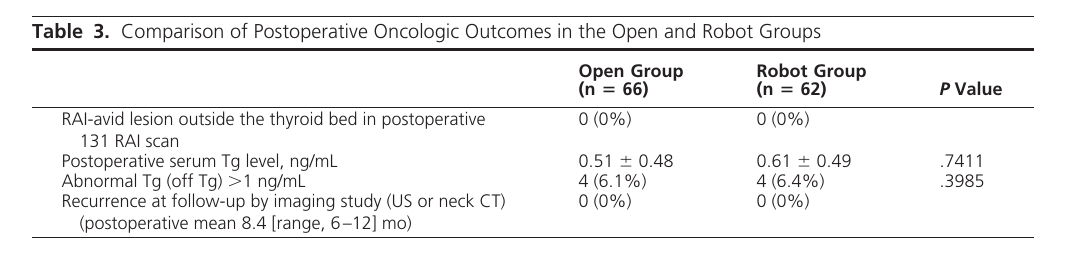


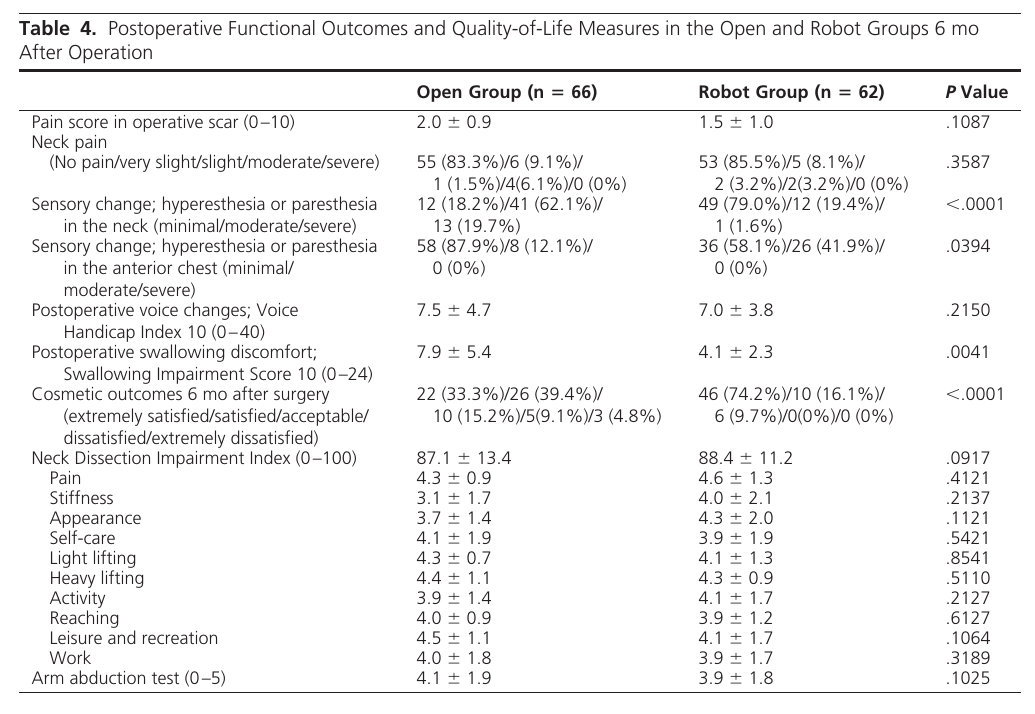

Supplement: S1 Dataset — (ZIP) [file pone.0298153.s003.zip › Data Set/3[7].docx]
