# Supplementary material for: A meta-analysis of unilateral axillary approach for robotic surgery compared with open surgery for differentiated thyroid carcinoma
Source: PLoS One. 2024 Apr 11;19(4):e0298153. doi: 10.1371/journal.pone.0298153 (PMC11008900; doi:10.1371/journal.pone.0298153)

**Title:** **A comparative North American experience of robotic thyroidectomy in a thyroid cancer population**

**Study design:** Cohort study Quality score:8

**Author**: Salem I. Noureldine

**Year**:2013

**Address**: USA Tulane University School of Medicine

**Surgeon**: Emad Kandil

**Surgery approach**: unilateral axillary approach

**Surgery time**:2009.12-2011.10

**Surgery extent**: Total thyroidectomy(TT) or Hemithyroidectomy

**Inclusion Criteria**: Differentiated thyroid cancer(DTC) without cervical lymph node dissection.

**Exclusion criteria**: Patients with cervical lymph node metastasis, distant metastases, and invasion to adjacent organs such as the trachea, recurrent laryngeal nerve (RLN), and esophagus required extensive surgery beyond thyroidectomy and therefore were excluded.

**Permanent recurrent laryngeal nerve injury**: more than 6 months

**Permanent hypoparathyroidism/hypocalcemia**: more than 6 months

**Follow-up**:12±2.2months


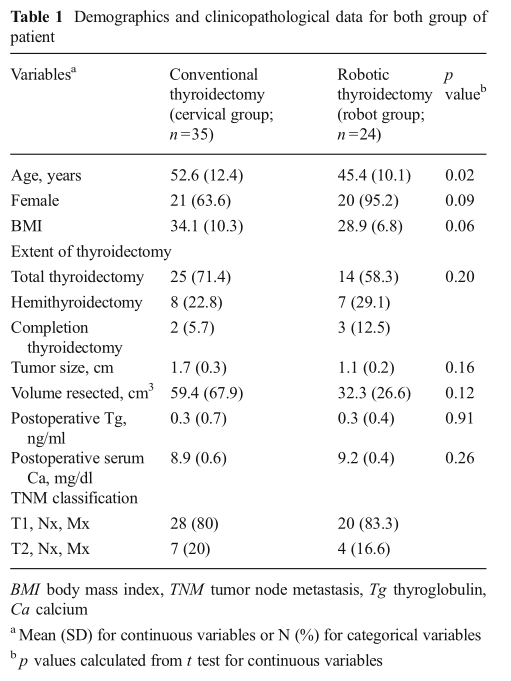


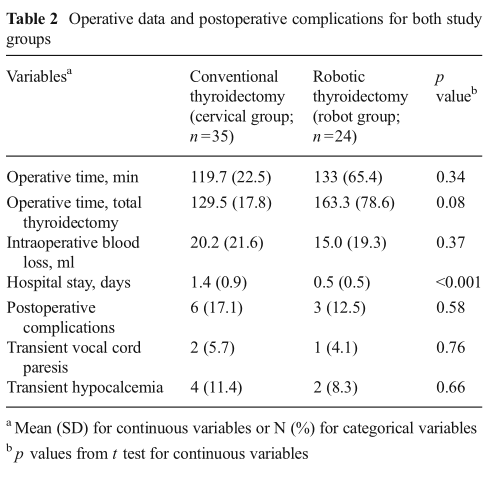

Supplement: S1 Dataset — (ZIP) [file pone.0298153.s003.zip › Data Set/4[8].docx]
