# Supplementary material for: A meta-analysis of unilateral axillary approach for robotic surgery compared with open surgery for differentiated thyroid carcinoma
Source: PLoS One. 2024 Apr 11;19(4):e0298153. doi: 10.1371/journal.pone.0298153 (PMC11008900; doi:10.1371/journal.pone.0298153)

**Title:** **A Comparison of Postoperative Pain After Conventional Open Thyroidectomy and Transaxillary Single-Incision Robotic Thyroidectomy: A Prospective Study**

**Study design:** non-randomized controlled study(NRS) Quality score:20

**Author**: Haeng Rang Ryu

**Year**:2013

**Address**: Korea Yonsei University thyroid cancer clinic

**Surgeon**: Woong Youn Chung

**Surgery approach**: unilateral axillary approach

**Surgery time**:2010.12-2011.05

**Surgery extent**: Total thyroidectomy(TT) with central compartment neck dissection(CCND)

**Inclusion Criteria**: (1)American Society of Anesthesiologist physical status I–II,(2) patient age of 20–60 years, (3) bilateral total thyroidectomy with ipsilateral central compartment node dissection, and (4) papillary thyroid carcinom.

**Exclusion criteria**: (1) a history of previous neck operations, (2) malignancy other than papillary thyroid carcinoma, (3) systemic disease such as renal failure, liver dysfunction, coronary artery occlusive disease, or asthma, (4) radiation history of neck or anterior chest. In robotic thyroidectomy, and (5) lesions located in the thyroid dorsal area (particularly at the tracheoesopheal groove) with posterior capsular invasion or extension to an adjacent structure.

**Permanent recurrent laryngeal nerve injury**:unclear

**Permanent hypoparathyroidism/hypocalcemia**: unclear

**Follow-up**: unclear


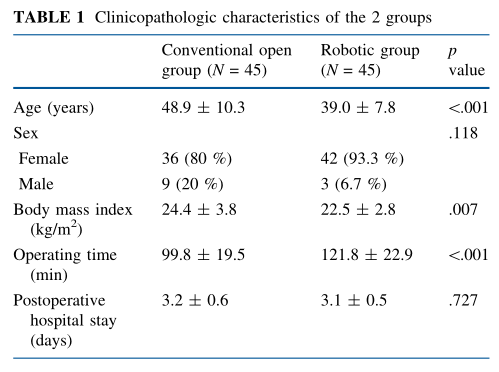


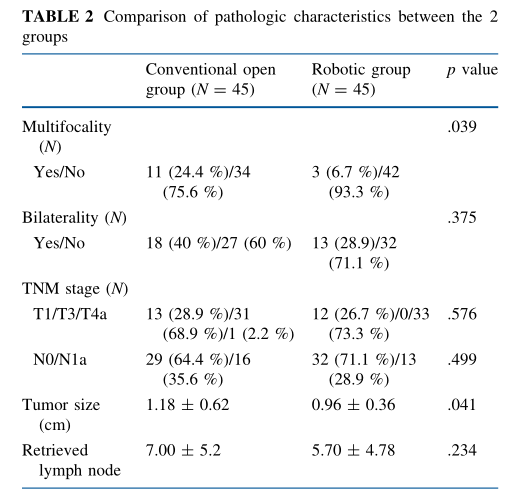


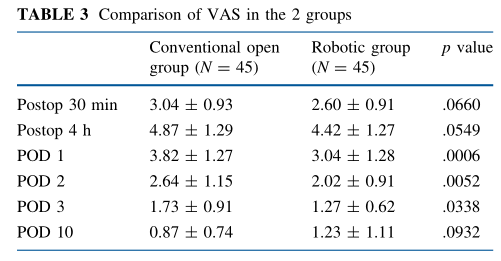

Supplement: S1 Dataset — (ZIP) [file pone.0298153.s003.zip › Data Set/5[9].docx]
