# Supplementary material for: A meta-analysis of unilateral axillary approach for robotic surgery compared with open surgery for differentiated thyroid carcinoma
Source: PLoS One. 2024 Apr 11;19(4):e0298153. doi: 10.1371/journal.pone.0298153 (PMC11008900; doi:10.1371/journal.pone.0298153)

**Title:** **Quality of Life After Robotic Thyroidectomy by a GaslessUnilateral Axillary Approach**

**Study design**: cross-sectional study Quality score: 18

**Author**: Chang Myeon Song

**Year**:2014

**Address**: Korea Hanyang University

**Surgeon**: Kyung Tae

**Surgery approach**: unilateral axillary approach

**Surgery time**:unclear

**Surgery extent**: Total thyroidectomy(TT) or lobectomy with central compartment neck dissection(CCND)

**Inclusion Criteria**: papillary thyroid carcinoma (PTC) patients having undergone thyroidectomy with or without postoperative radioactive iodine (RAI) ablation, no recurrence or persistent disease at the time of evaluation, and no further planned therapy for thyroid cancer except thyroid-stimulating hormone suppressive therapy with levothyroxine replacement.

**Exclusion criteria**: concomitant lateral neck dissection, distant metastasis, extensive extrathyroidal extension necessitating resection of the aerodigestive tract or recurrent laryngeal nerve, comorbidity requiring medical or surgical treatment that could influence health-related quality of life (HRQOL), or history of neck surgery or irradiation

**Permanent recurrent laryngeal nerve injury**: more than 6 months

**Permanent hypoparathyroidism/hypocalcemia**: unclear

**Follow-up**:12 months


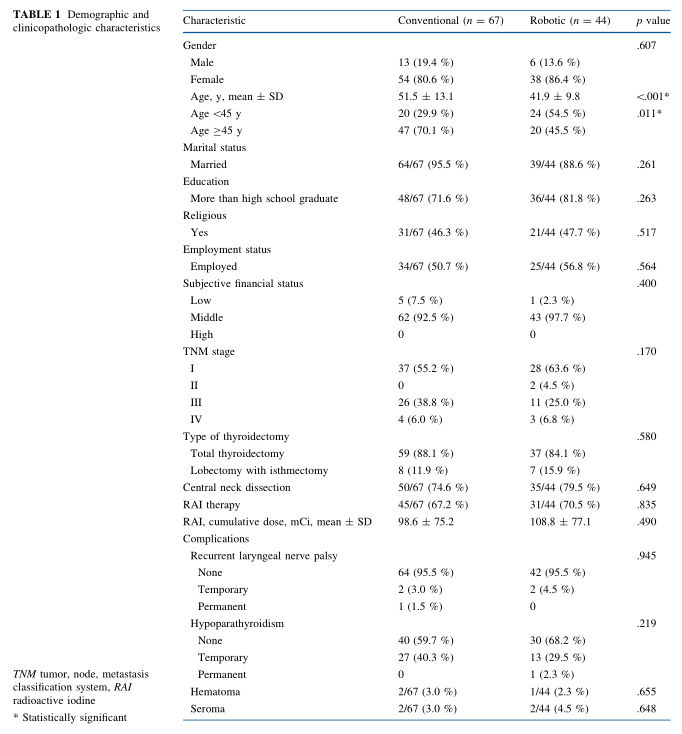


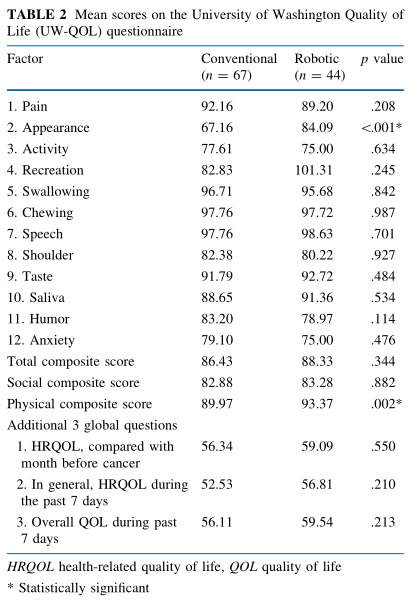


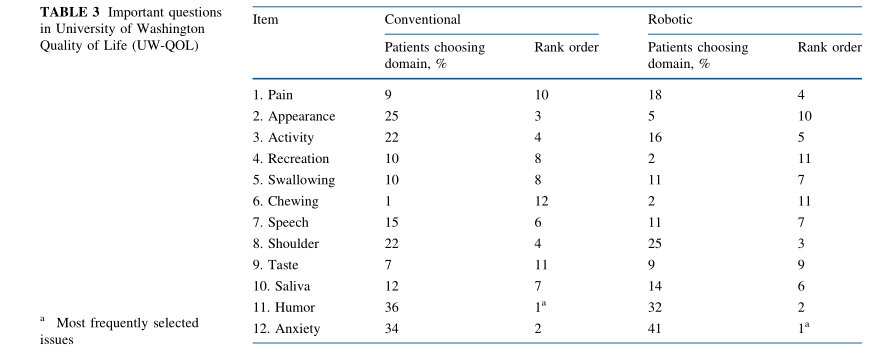

Supplement: S1 Dataset — (ZIP) [file pone.0298153.s003.zip › Data Set/6[10].docx]
