# Supplementary material for: A meta-analysis of unilateral axillary approach for robotic surgery compared with open surgery for differentiated thyroid carcinoma
Source: PLoS One. 2024 Apr 11;19(4):e0298153. doi: 10.1371/journal.pone.0298153 (PMC11008900; doi:10.1371/journal.pone.0298153)

**Title:** **Long-term oncologic outcome of robotic versus open total thyroidectomy in PTC: a case-matched retrospective study**

**Study design**: Cohort study with propensity score matching Quality score: 8

**Author**: Seul Gi Lee

**Year**:2016

**Address**: Korea Yonsei Cancer Center

**Surgeon**: Woong Youn Chung

**Surgery approach**: unilateral axillary approach

**Surgery time**:2007.11-2010.02

**Surgery extent**: Total thyroidectomy(TT) with central compartment neck dissection(CCND)

**Inclusion Criteria**: patients with papillary thyroid cancer (PTC) who underwent TT

with central compartment node dissection (CCND)

**Exclusion criteria**: PTC patients with extensive extrathyroidal invasion, including major vessels, trachea, esophagus or recurrent laryngeal nerve; lateral cervical LN and distant metastases; and previous neck surgery, were excluded.

**Permanent recurrent laryngeal nerve injury**: unclear

**Permanent hypoparathyroidism/hypocalcemia**: unclear

**Follow-up**:74.2 months


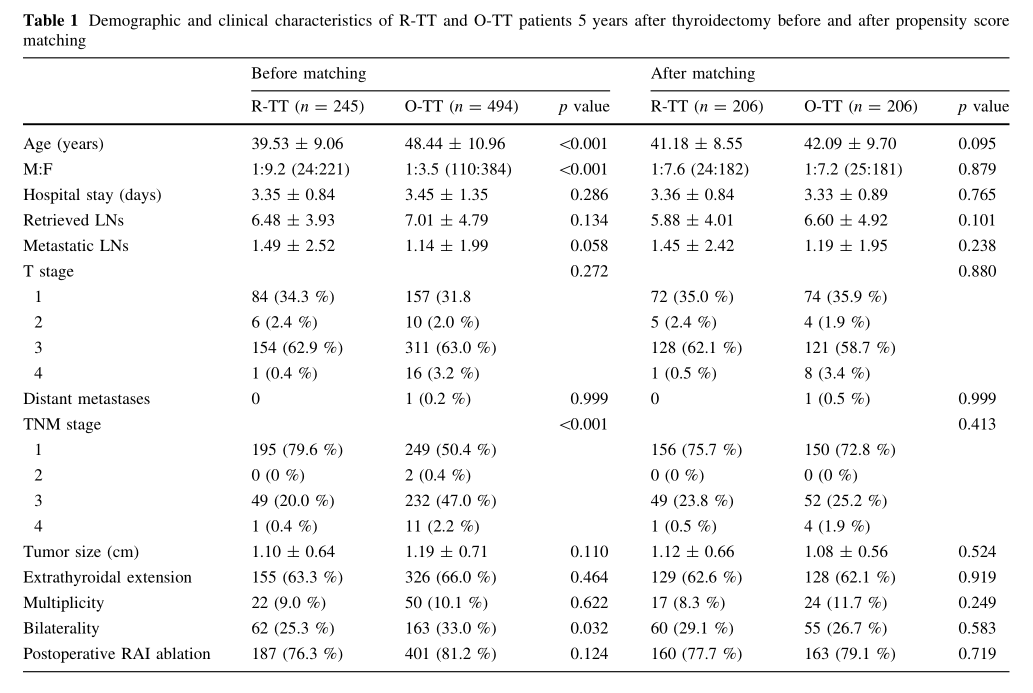


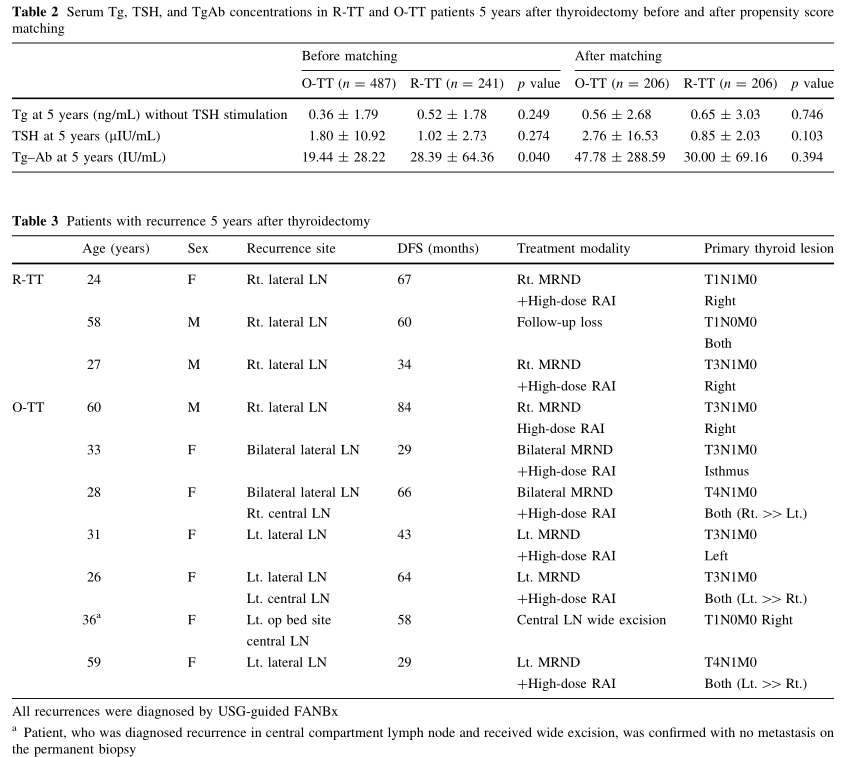

Supplement: S1 Dataset — (ZIP) [file pone.0298153.s003.zip › Data Set/7[11].docx]
