# Supplementary material for: A meta-analysis of unilateral axillary approach for robotic surgery compared with open surgery for differentiated thyroid carcinoma
Source: PLoS One. 2024 Apr 11;19(4):e0298153. doi: 10.1371/journal.pone.0298153 (PMC11008900; doi:10.1371/journal.pone.0298153)

**Title:** **Comparison of Robotic versus Conventional Selective Neck Dissection and Total Thyroidectomy for Papillary Thyroid Carcinoma**

**Study design**: Cohort study Quality score: 8

**Author**: Chang Myeon Song

**Year**:2016

**Address**: Korea Hanyang University

**Surgeon**: Kyung Tae

**Surgery approach**: unilateral axillary approach or unilateral axillo-breast approach

**Surgery time**:2009.11-2013.09

**Surgery extent**: Total thyroidectomy(TT) with central compartment neck dissection(CCND) and modified radical neck dissections (MRND)

**Inclusion Criteria**: cN1b PTC patients with metastatic LNs≤4 cm in size in the lateral compartment suspected by ultrasonography and/or computed tomography or confirmed by fine-needle aspiration cytology

**Exclusion criteria**: large conglomerated metastatic LNs in the central or lateral compartment, LN metastasis with extensive invasion of surrounding structures in preoperative ultrasonography and/or computed tomography, tumors with extensive extrathyroidal extension, recurred tumors, distant metastases, and a history of neck irradiation or surgery.

**Permanent recurrent laryngeal nerve injury**: more than 6 months

**Permanent hypoparathyroidism/hypocalcemia**: more than 6 months

**Follow-up**: 29±11.4 months, 34.8±14.5 months


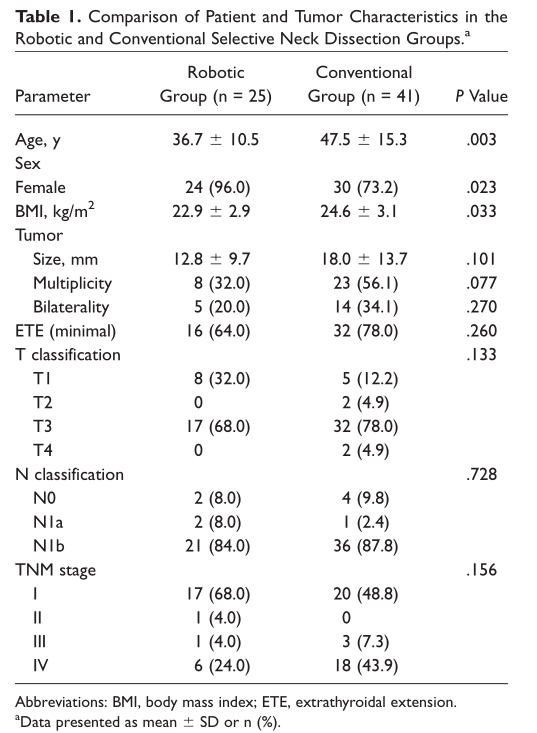


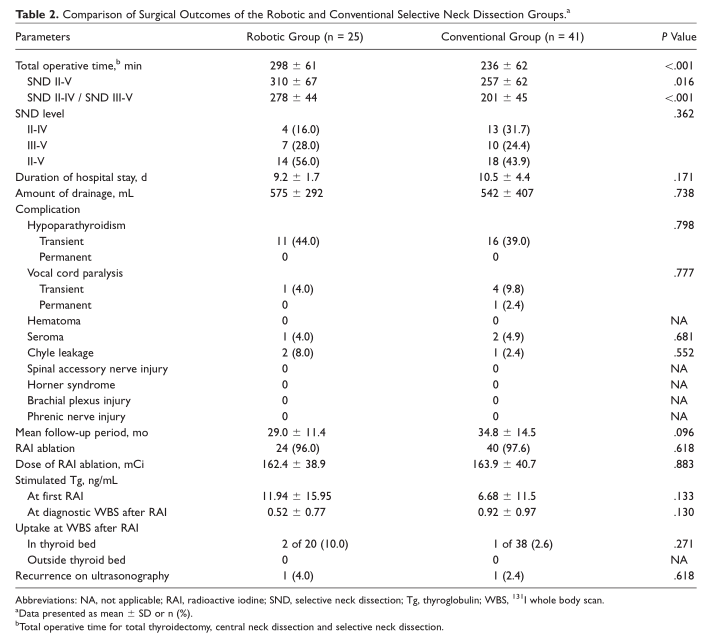


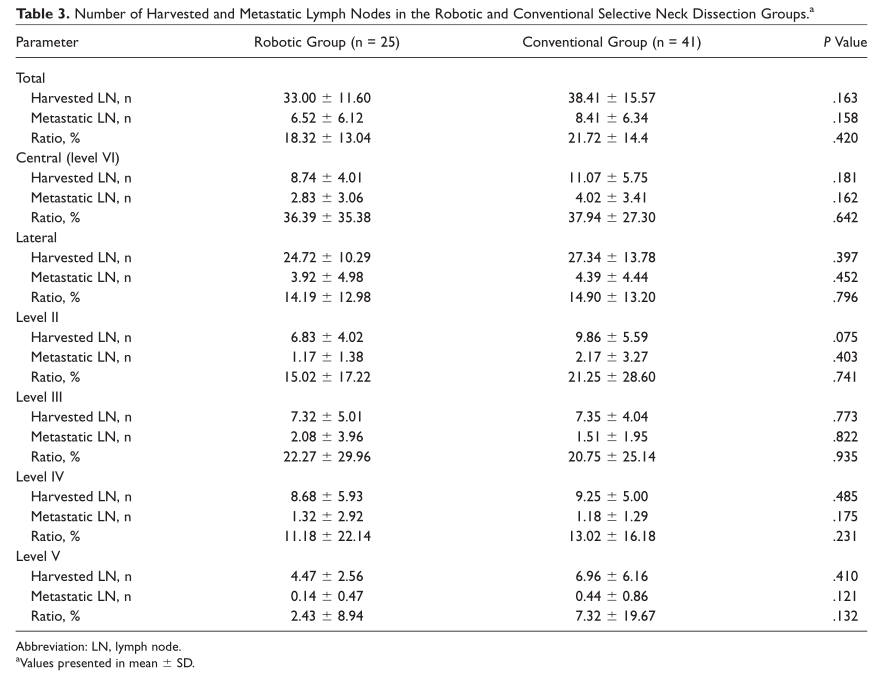


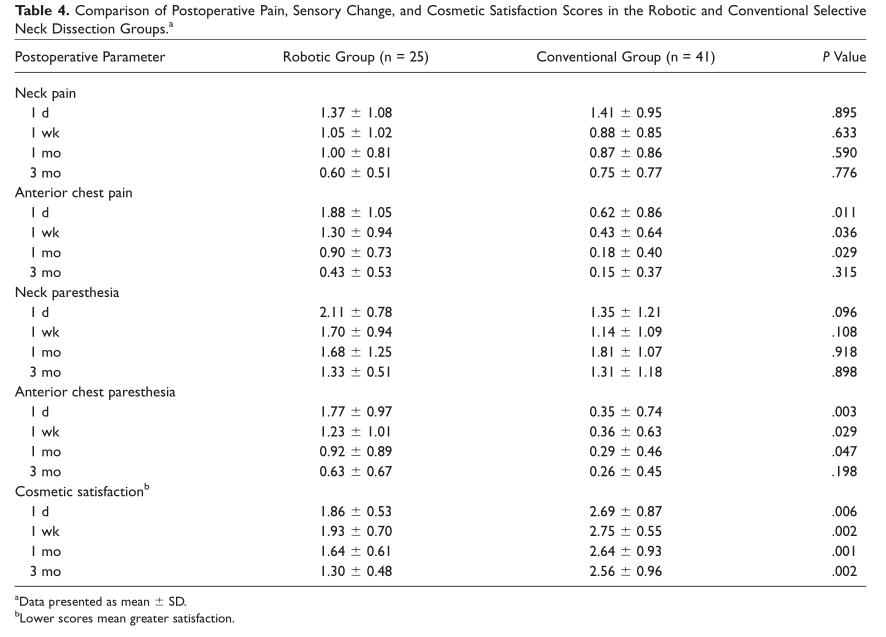

Supplement: S1 Dataset — (ZIP) [file pone.0298153.s003.zip › Data Set/8[12].docx]
