# Supplementary material for: A meta-analysis of unilateral axillary approach for robotic surgery compared with open surgery for differentiated thyroid carcinoma
Source: PLoS One. 2024 Apr 11;19(4):e0298153. doi: 10.1371/journal.pone.0298153 (PMC11008900; doi:10.1371/journal.pone.0298153)

**Title:** **Oncologic outcomes of robotic thyroidectomy: 5-year experience with propensity score matching**

**Study design**: Cohort study with propensity score matching Quality score: 9

**Author**: Kyung Tae

**Year**:2016

**Address**: Korea Hanyang University

**Surgeon**: Kyung Tae

**Surgery approach**: unilateral axillary approach or unilateral axillo-breast approach

**Surgery time**:2008.10-2014.02

**Surgery extent**: Total thyroidectomy(TT) or lobectomy with central compartment neck dissection(CCND)

**Inclusion Criteria**: PTC patients included tumors of less than 4 cm with or without minimal extrathyroidal extension (ETE), or metastatic lymph nodes less than 4 cm in the central or lateral compartment on preoperative ultrasonography (US) and/or computed tomography (CT).

**Exclusion criteria**: PTC patients with gross maximal ETE, multiple conglomerated lymph node metastases with extensive invasion of surrounding structures in the central or lateral compartment, and distant metastasis or with a history of neck or thyroid surgery or irradiation.

**Permanent recurrent laryngeal nerve injury**: more than 6 months

**Permanent hypoparathyroidism/hypocalcemia**: more than 6 months

**Follow-up**: 43.6 months


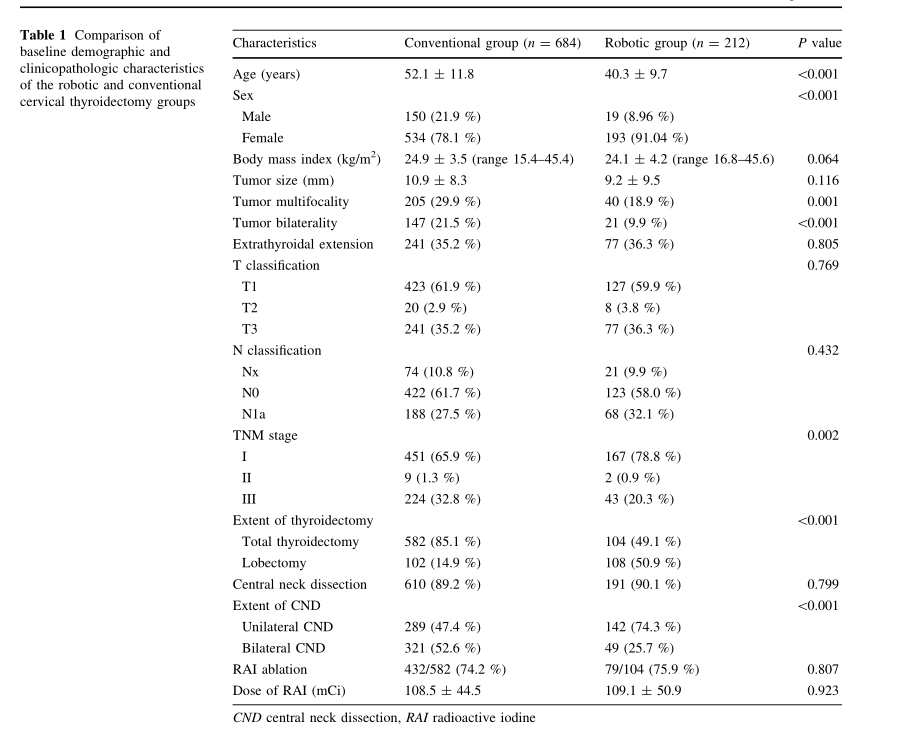


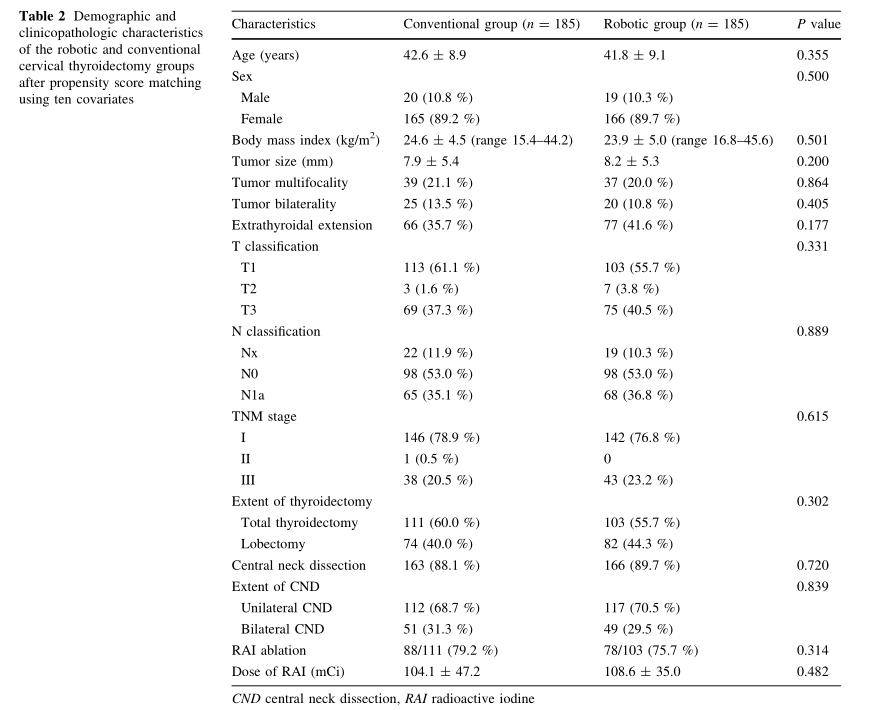


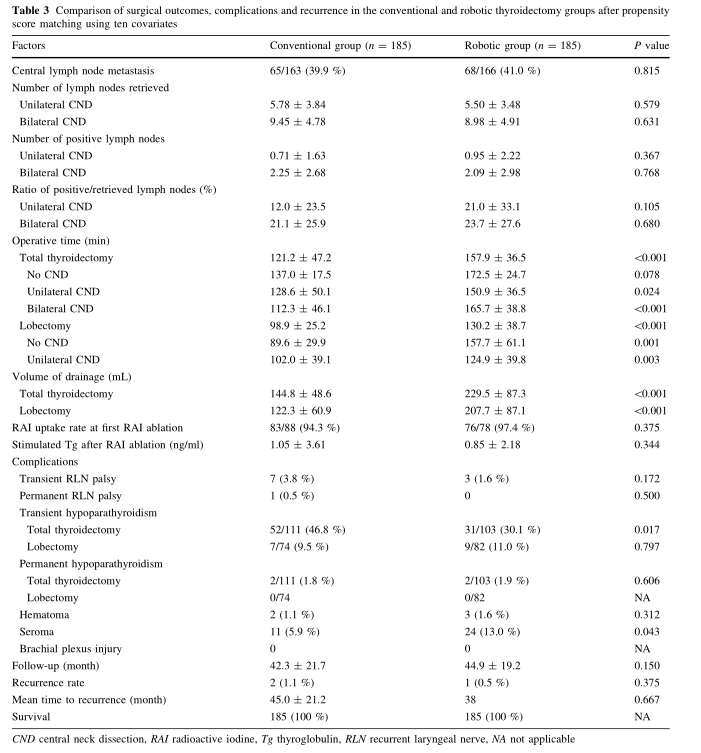

Supplement: S1 Dataset — (ZIP) [file pone.0298153.s003.zip › Data Set/9[13].docx]
